# Lipid Annotation by Combination of UHPLC-HRMS (MS), Molecular Networking, and Retention Time Prediction: Application to a Lipidomic Study of In Vitro Models of Dry Eye Disease

**DOI:** 10.3390/metabo10060225

**Published:** 2020-05-29

**Authors:** Romain Magny, Anne Regazzetti, Karima Kessal, Gregory Genta-Jouve, Christophe Baudouin, Stéphane Mélik-Parsadaniantz, Françoise Brignole-Baudouin, Olivier Laprévote, Nicolas Auzeil

**Affiliations:** 1 Sorbonne Université UM80, INSERM UMR 968, CNRS UMR 7210, Institut de la Vision, IHU ForeSight, 75006 Paris, France; romain.magny@inserm.fr (R.M.); karima.kessal@inserm.fr (K.K.); cbaudouin@15-20.fr (C.B.); stephane.melik-parsadaniantz@inserm.fr (S.M.-P.); fbaudouin@15-20.fr (F.B.-B.); 2 UMR CNRS 8038 CiTCoM, Chimie Toxicologie Analytique et Cellulaire, Université de Paris, Faculté de Pharmacie, 75006 Paris, France; anne.regazzetti@parisdescartes.fr (A.R.); gregory.genta-jouve@parisdescartes.fr (G.G.-J.); olivier.laprevote@aphp.fr (O.L.); 3 Centre Hospitalier National d’Ophtalmologie des Quinze-Vingts, IHU ForeSight, 75006 Paris, France; 4 Laboratoire Ecologie, Evolution, Interactions des Systèmes Amazoniens (LEEISA), USR 3456, Université De Guyane, CNRS Guyane, 97300 Cayenne, French Guiana, France; 5 Hôpital Ambroise Paré, AP-HP, Université Versailles Saint-Quentin-en-Yvelines, 92100 Boulogne-Billancourt, France; 6 Hôpital Européen Georges Pompidou, AP-HP, Service de Biochimie, 75006 Paris, France

**Keywords:** lipidomic, liquid chromatography, tandem mass spectrometry, molecular network, dry eye disease, hyperosmolarity

## Abstract

Annotation of lipids in untargeted lipidomic analysis remains challenging and a systematic approach needs to be developed to organize important datasets with the help of bioinformatic tools. For this purpose, we combined tandem mass spectrometry-based molecular networking with retention time (t_R_) prediction to annotate phospholipid and sphingolipid species. Sixty-five standard compounds were used to establish the fragmentation rules of each lipid class studied and to define the parameters governing their chromatographic behavior. Molecular networks (MNs) were generated through the GNPS platform using a lipid standards mixture and applied to lipidomic study of an *in vitro* model of dry eye disease, *i.e.*, human corneal epithelial (HCE) cells exposed to hyperosmolarity (HO). These MNs led to the annotation of more than 150 unique phospholipid and sphingolipid species in the HCE cells. This annotation was reinforced by comparing theoretical to experimental t_R_ values. This lipidomic study highlighted changes in 54 lipids following HO exposure of corneal cells, some of them being involved in inflammatory responses. The MN approach coupled to t_R_ prediction thus appears as a suitable and robust tool for the discovery of lipids involved in relevant biological processes.

## 1. Introduction

Over the last decade, lipids have become a major research topic and are now recognized as key biological compounds displaying various roles in cell functions. They include the coordination of bio-membrane structures, intra- and extra-cellular communication, metabolic efficiency, and signaling cascades, all of which are critical for cell functionality [1]. Disruption of lipid homeostasis is now recognized to be involved in numerous pathologies such as cancer, diabetes, neurodegenerative disorders or chronic inflammatory diseases [2,3]. Lipids, especially phospholipid and sphingolipid classes, are central in both inflammatory and cell death processes [4,5]. Arachidonic acid, mainly originating from the cleavage of phospholipids, is, for example, widely recognized as a pro-inflammatory fatty acid [6]. Besides, ceramides, a sphingolipid subclass, mediate apoptosis through a caspase-3 dependent mechanism and inflammation through the release of cytokines such as IL-1ß or IL-6 [7].

Nevertheless, lipids encompass a tremendous number of molecular species exhibiting a wide variety of structures. Indeed, the cellular lipidome includes numerous subclasses of sphingolipids, phospholipids, glycerolipids, sterol lipids, and lipid metabolites. Lipidomics, the comprehensive analysis of lipids in biological systems, remains challenging and must involve not only efficient analytical techniques but also appropriate sample processing and integrative computational approaches [8]. Electrospray ionization (ESI) mass spectrometry (MS), either through a shotgun approach or hyphenated to liquid chromatography (LC), has become the gold standard of lipidome study [8,9]. Indeed, lipidomic analysis using an infusion approach represents a useful strategy to easily access a large part of the lipidome [10,11]. Nevertheless, despite fruitful applications, this approach still suffers from ion suppression which limits the analysis of low abundant lipid species [10,12]. In contrast, lipidomic analysis using liquid chromatography hyphenated to mass spectrometry includes a separation step reducing ion suppression and, therefore, improves detection of low abundant lipid species through an increase in sensitivity [9,11,13]. Recent advances highlight that ion mobility, combined to chromatography and mass spectrometry, represents an additional source of information in the case of lipid identification [14,15]. Nevertheless, whatever the benefit of these techniques, the fact is that, in the context of a lipidomic analysis, the large amount of data to be processed requires the development of new data processing approaches, in particular, to simplify the annotation of lipid species while maintaining a high degree of reliability.

In the course of a lipidomics study, the reliable identification of the numerous lipid species detected represents a rigorous and demanding task. Lipids identification is performed taking into account four analytical features: retention time (t_R_), accurate precursor ion *m/z* value, isotopic ratio, and MS/MS data through comparison to reference compounds [16]. In the case of phospholipids, identification deals with the determination of the polar head group and the length of the acyl chains and of their sn_1_/sn_2_ location on the glycerol moiety. It must be emphasized that such identification is biologically strongly relevant inasmuch as the nature and position of acyl chains depend on the homeostatic balance between biosynthesis rates, remodeling, and degradation and also reflect the extent of the pool of fatty acids available.

To elucidate the structure of unknown compounds, bioinformatics tools are strongly valuable. Among them, molecular networks (MNs) have recently been proposed, historically in the field of plant secondary metabolites, to identify compounds of biotechnological interest or exhibiting promising pharmacological activity [17,18,19,20]. Molecular networks are a computational strategy aimed at organizing and visualizing hundreds of molecules using their MS/MS spectra in accordance to their similarities through the assumption that structurally related molecules display similar product ion spectra [21]. The structural similarity of a set of compounds is visualized in a network which may be generated through online platforms such as GNPS or MetGem [21,22].

Taking into account the LC retention time represents an important benefit for the identification of compounds, as it makes it possible to discriminate isobaric compounds [23]. For this purpose, a pre-processing step of the LC-MS/MS data is required; it may be performed using software such as MzMine 2 [24]. Furthermore, based on structural properties, retention time can easily be predicted with good reliability, and t_R_ values have previously been used to support the identification of numerous metabolite classes, especially lipids [25,26].

The aim of our study was to propose a new approach for the rapid and reliable structural annotation of phospholipids and sphingolipids species using molecular networking and t_R_ prediction. Using 65 commercial lipid standards, we first determined the collision energy conditions required to achieve the fragmentation patterns appropriate to the structural annotation of lipids. To support lipids annotation, commercial standards were further used to define the relationship between lipid structure and t_R_. Unknown lipids were then identified based on their exact mass measurements and MS/MS fragmentation through molecular networking and t_R_ values. 

This approach was used to perform a lipidomic analysis of human corneal epithelial cells exposed to hyperosmolarity (HO)—an *in vitro* model of dry eye disease (DED) [27,28]. Dry eye disease, a chronic multifactorial inflammatory pathology, is characterized by alteration of tear film, cell damage, and inflammation of the ocular surface [29,30]. This very common ocular pathology is also characterized by HO, one of the core mechanisms of DED [31]. Disruption of lipid homeostasis, known to be involved in inflammation and the cell death process, may also be a key feature in the pathophysiology of DED [32]. Thanks to MNs and t_R_ prediction, our lipidomic approach allowed annotation of 150 unique lipid species and highlights homeostasis disruption of 54 lipid species. Several of them are involved in inflammation and cell death.

## 2. Results and Discussion 

**Figure 1:**
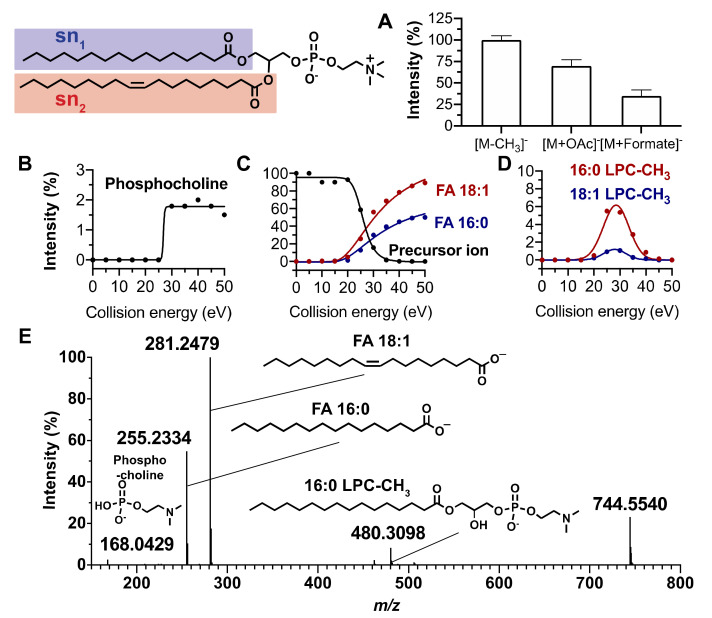
MS characteristics of PC (16:0/18:1). (**A**) Relative intensities of [M−CH_3_]^−^, [M+OAc]^−^, and [M+HCOO]^−^ ions. (**B**–**D**) Fragmentation patterns of the [M−CH_3_]^−^ ion formed from PC (16:0/18:1) under negative ionization conditions: relative intensities versus collision energy of (**B**) polar head group, (**C**) fatty acyl side chains, and (**D**) demethylated lysophosphatidylcholine ions. (**E**) Tandem mass spectrum of PC (16:0/18:1) [M−CH_3_]^−^ ion obtained by collision energy ramping from 20 to 40 eV. Blue and red colors relate to the sn_1_ and sn_2_ positions, respectively.

**Figure 2:**
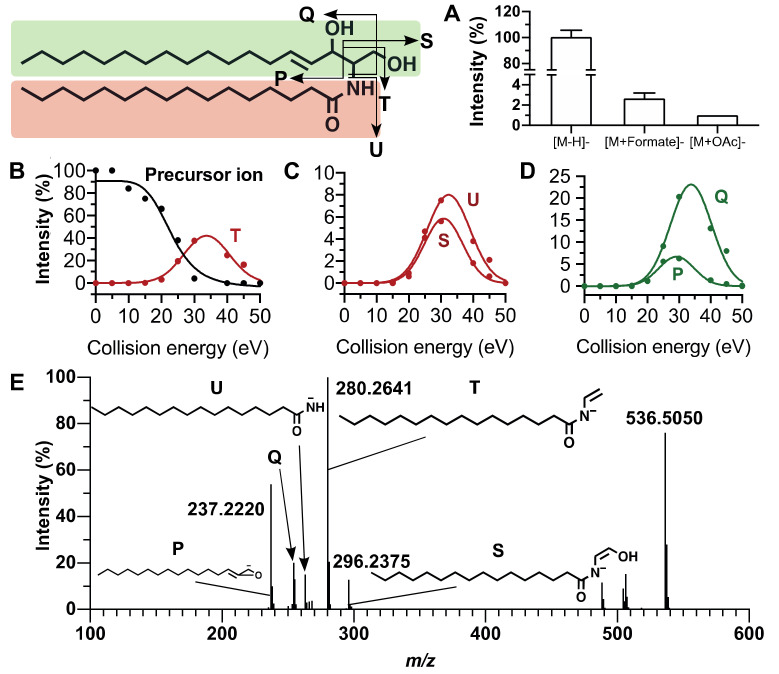
MS characteristics of Cer (d18:1/16:0). (**A**) Relative intensities of [M−H]^−^, [M+OAc]^−^, and [M+HCOO]^−^ ions. (**B**–**D**) Fragmentation patterns of the [M−H]^−^ ion formed from Cer (d18:1/16:0) under negative ionization conditions: relative intensities versus collision energy of (**B**) precursor ion, (**C**) fatty acyl side chains, and (**D**) sphinganine ions. (**E**) Tandem mass spectrum of Cer (d18:1/16:0) [M−H]^−^ ion obtained by collision energy ramping from 20 to 40 eV. Product ions: T (*m/z* 280.2646), U (*m/z* 254.2486), and S (*m/z* 296.259) are indicative of the fatty acyl chains; Q (*m/z* 263.2379) and P (*m/z* 237.2225) are indicative of the sphingosine moiety. Product ions are labelled according to Reference [37]. Green and red colors relate to sphingosine and fatty acyl position, respectively.

Reliable annotation of unknown compounds using MN highly depends on the quality of the acquired MS/MS spectra [33]. For this purpose, we performed a set of MS/MS experiments for which collision energy was increased step by step in order to optimize the diagnostic fragment ion intensities. Phospholipid annotation needs the presence on the MS/MS spectra of product ions corresponding to the polar head group, the fatty acyl side chains and of the precursor ion. For sphingolipid annotation, fragments corresponding to the sphinganine base moiety and fatty acyl side chain must be detected on MS/MS spectra. Figure 1 and Figure 2 exhibit the main MS characteristics related to PC (16:0/18:1) and Cer (d18:1/16:0), respectively.

### 2.1. Fragmentation Patterns of Phospholipids

In the negative ion mode, phosphatidylcholine (PC) are mainly detected as [M−CH_3_]^−^ ions corresponding to an in-source loss of a methenium and, to a less extent, as formiate ([M+HCOO]^−^) and acetate ([M+CH_3_COO]^−^) adducts (Figure 1A). In our study, annotation of a PC was based on the detection of six diagnostic product ion peaks in the MS/MS spectrum of [M−CH_3_]^−^.

In the example shown in Figure 1 related to PC (16:0/18:1), the precursor ion was observed at *m/z* 744.5540. At low mass, a peak at *m/z* 168.0423 corresponded to the deprotonated demethylated phosphocholine ion formed at a 25 eV collision energy (Figure 1B). At higher mass, the peaks at *m/z* 255.2334 and *m/z* 281.2479 were assigned to oleate and palmitate and exhibited an increased intensity from 20 to 50 eV collision energy (Figure 1C). Two other key product ions at *m/z* 480.3098 and *m/z* 506.3256 corresponded to demethylated lysophosphatidylcholine LPC (16:0) and LPC (18:1) ions, respectively. They were detected from 20 to 40 eV collision energy with a maximum intensity at 30 eV (Figure 1D). A collision energy ramp between 20 and 40 eV thus appeared to be suitable to obtain the six diagnostic ions with sufficient sensitivity and mass accuracy (△ < 10 ppm) (Figure 1E). The ions used to identify 10 standard PC species are compiled in Table S3.

Interestingly, while in the positive ion mode, the phosphocholine product ion at *m/z* 184.0733 allows for the highly sensitive detection of PC, and the negative ion mode is essential to perform fatty acyl chains identification [34]. This ionization mode was also successfully applied to identify the fatty acyl chains of other phospholipid subclasses, namely, phosphatidylethanolamine (PE), phosphatidylinositol (PI), phosphatidylserine (PS), phosphatidylglycerol (PG), and phosphatidic acid (PA). It also proved useful to locate the fatty acyl chains on the sn_1_ and sn_2_ positions of the glycerol core. Indeed, for PC, PE, and PG species, the intensity of the carboxylate ion peak corresponding to the fatty acid at the sn_2_ position was always significantly higher than that at sn_1_. This is in agreement with previously published data [35]. For example, Figure 1C shows an oleate peak at sn_2_ more intense than the sn_1_ palmitate in the whole collision energy range. Similarly, the fragment corresponding to demethylated LPC (16:0) formed by the loss of the sn_2_ oleate from the precursor ion was more intense than the demethylated LPC (18:1) arising from the loss of the sn_1_ palmitate (Figure 1D). For the lipids belonging to the PS, PI, and PA subclasses, the acyl group at sn_1_ always led to the more intense product ion peak (Figures S2 and S3) [35]. However, it is noteworthy that the relative intensity of carboxylate product ions displayed in the MS/MS spectra only provided information on fatty acids sn_1_ and sn_2_ locations regarding the major regio-isomer. Indeed, the presence in the mixture of a minor amount of the other regio-isomer cannot be excluded. Ensuring it, would need to build a calibration curve using the two pure regio-isomers [36]. In this study, we thus report what is likely to be the major regio-isomer.

The polar head groups were identified owing to the presence of specific fragment ions such as *m/z* 168.0428 and *m/z* 224.0694 for PC, *m/z* 140.0113 and *m/z* 196.0380 for PE, *m/z* 227.0326 for PG, and *m/z* 241.0119 for PI. For PS, an abundant and specific serine loss (87.0326 Da) was observed in the MS/MS spectra of the deprotonated molecules [M−H]^−^ together with a glycerophosphate ion at *m/z* 152.9958, whereas PA only led to a glycerophosphate ion. Table S3 in the Supplementary Materials lists the diagnostic ions for the 65 phospholipid standard species included in our study.

### 2.2. Fragmentation Patterns of Sphingolipids

Ceramides (Cer) are based on a sphinganine backbone amidated by a fatty acyl side chain. Ceramides are also building blocks of more complex sphingolipids such as hexosyl ceramide (HexCer) or sphingomyelins (SM). Under negative ionization conditions, Cer were mainly detected as [M−H]^−^ and, to a lesser extent, as acetate ([M+CH_3_COO]^−^) and formate ([M+HCOO]^−^) adducts (Figure 2A). As for phospholipids, annotation of individual ceramides was based on the detection of six diagnostic ions which made it possible to determine the double bound number of the sphinganine backbone as well as the FA side chain length. Fatty acyl chain identification was performed thanks to the MS/MS spectra of the [M−H]^−^ ion at collision energies of 20 eV and more (Figure 2B,C).

In the example shown at Figure 2, product ions labelled T (*m/z* 280.2646), U (*m/z* 254.2486) and S (*m/z* 296.259) are indicative of the fatty acyl chain (Figure 2B,C). The sphingosine moiety is characterized by the product ions Q at *m/z* 263.2379 and P at *m/z* 237.2225 formed at collision energies higher than 20 eV (Figure 2D). The [M−H]^−^ precursor ion and its product ions [M−H−H_2_O]^−^ and [M−H−2H_2_O]^−^ are detected in the high mass region (Figure 2E). As exemplified by Cer (d18:1/16:0), a collision energy ramping from 20 to 40 eV proved suitable to detect the six diagnostic ions with good sensitivity and mass accuracy (△ < 2 ppm), allowing an easy annotation of Cer species. MS/MS spectra of Cer displayed more intense product ion peaks than PC therefore resulting in a better accuracy of *m/z* values.

### 2.3. Retention Time Prediction

**Figure 3:**
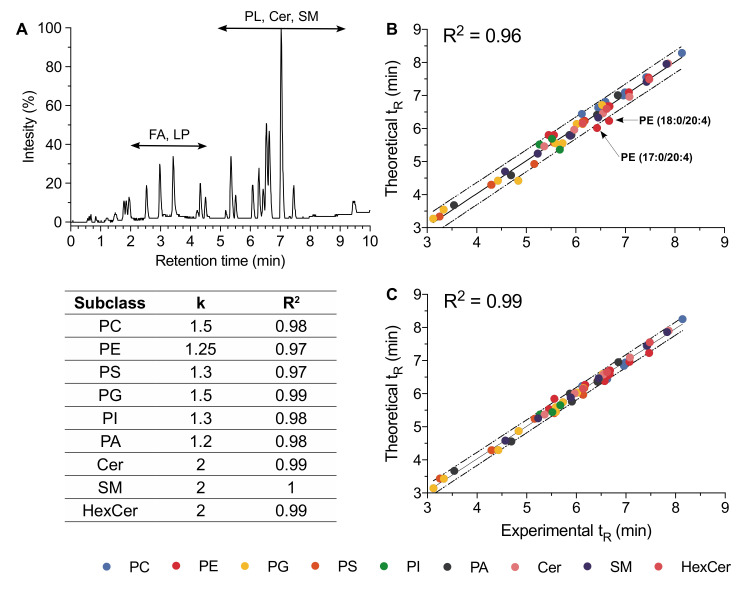
Retention time prediction model based on equivalent carbon number (ECN) of nine subclasses of phospholipids and sphingolipids. (**A**) UHPLC-HRMS chromatogram of a commercial standard lipid mixture in the negative ion mode. (**B**) Theoretical *t*_R_ plotted against experimental *t*_R_ for standard lipid mixture before and (**C**) after k value fitting. A linear (phospholipids) and polynomial (sphingolipids) regression model was used. Dotted lines represent the 95% confidence interval displaying a relative error which reached 15% on the linear regression graph (B) (k = 2) and did not exceed 5% on the linear regression graph (C). Table: Parameters of relationship between ECN and experimental t_R_ determined using a standard lipid mixture. The general expression of ECN is ECN = NC - k × DB where NC and DB are the total number of carbons and the number of double bonds, respectively. PC: phosphatidylcholine, PE: phosphatidylethanolamine, PG: phosphatidylglycerol, PS: phosphatidylserine, PI: phosphatidylinositol, PA: phosphatidic acid, Cer: ceramide, SM: sphingomyelin, HexCer: hexosyl ceramide.

A typical UHPLC-ESI-MS negative ion mode chromatogram of a mixture of 65 lipid standards representative of the nine studied subclasses is displayed in Figure 3A. In reversed-phase liquid chromatography, an elution of lipids is closely related to the fatty acyl chain lengths, and this property has been widely used in the frame of lipidomic analyses [38,39]. Under our conditions, the FA and lysophospholipids were firstly eluted for 6 min followed by Cer and phospholipids (*i.e.*, PE, PI, PG, and PS) between 6 and 9 min (Figure 3A). Furthermore, in the case of phospholipids, the chromatographic behavior was also dependent on the polar head group, the elution order being for a given fatty acyl chain pattern as follows: PI, PG, PS, PC, and PE (Figure S1). Retention time may thus be considered as a valuable analytical feature helpful in confirming annotation or in highlighting misannotation. However, this requires a robust chromatographic system able to deliver stable and reliable retention times. In our hands, RP-UHPLC operating with a reduced particle size (1.7 µm), associated to a column temperature of 50 °C, and optimized elution conditions provided chromatograms with peak widths lower than 20 s and highly reproducible retention times (Table S2).

In order to reinforce lipid annotation using t_R_, we first used the commercial lipid standard to build a t_R_ predictive model based on the equivalent carbon numbers (ECNs). The general expression of ECNs is ECN = NC – k × DB, where NC and DB are the total number of carbons and the number of double bonds, respectively. For lipid species, k = 2 is usually applied [40]. 

In the case of phospholipids, especially when containing polyunsaturated fatty acid (PUFA), the linear t_R_ prediction model using k = 2 was fairly poor (Figure 3B). To improve the t_R_ prediction model, we plotted ECN values with experimental t_R_ allowing, next to an appropriate fitting step, to determine more accurate k values. New k values calculated for the different investigated phospholipid subclasses thus improved the linear correlation between ECN and t_R_ (Figure 3C). For example, the difference between the theoretical and experimental t_R_ values for PE (17:0/20:4) was 13% for k = 2 and decreased to 0% using k = 1.25.

In the case of sphingolipids, the linear t_R_ prediction model with k = 2 was suitable for species whose fatty acyl chains did not exceed 22 carbon atoms. However, regarding this lipid class, a polynomial curve led to a better t_R_ prediction model than a linear one. The selection of the polynomial degree was based on the value of the correlation coefficient using a tolerance of 1/100 (*R*^2^ > 0.99). For the three investigated sphingolipid subclasses, a quadratic function was finally retained.

Based on this improved fitting step, we predicted more accurately the theoretical t_R_ for all the commercial standards lipids including six phospholipid subclasses and three sphingolipid subclasses (Figure 3C,D). Indeed, the difference between the experimental and theoretical t_R_ values did not exceed 5%, whatever the commercial standard lipid. Consequently, an uncertainty of 5% was retained when t_R_ was used in the purpose to support the annotation of unknown lipids from HCE cell extract and also to discriminate two isobaric lipid species as exemplified later in the text (see Section 2.6).

### 2.4. Instrument Stability

The stability of the UHPLC-ESI-MS/MS system was assessed for both MS and chromatography. For this purpose, exact mass measurements were performed on the standard lipid species. High-resolution mass measurements led to a mass accuracy better than 5 ppm for whichever standard lipid species under consideration (Table S2) and the chromatographic system delivered t_R_ values with a deviation within a 3-day period not exceeding 3% (Table S2).

### 2.5. Lipidic Networking of Human Corneal Epithelial Cells

Although untargeted analysis by MS constitutes a relevant and powerful tool to characterize a cell lipidome, the annotation of the lipids of interest remains a real challenge. Fortunately, phospholipids and sphingolipids display structural characteristics which make their identification suitable through molecular networking. 

**Figure 4:**
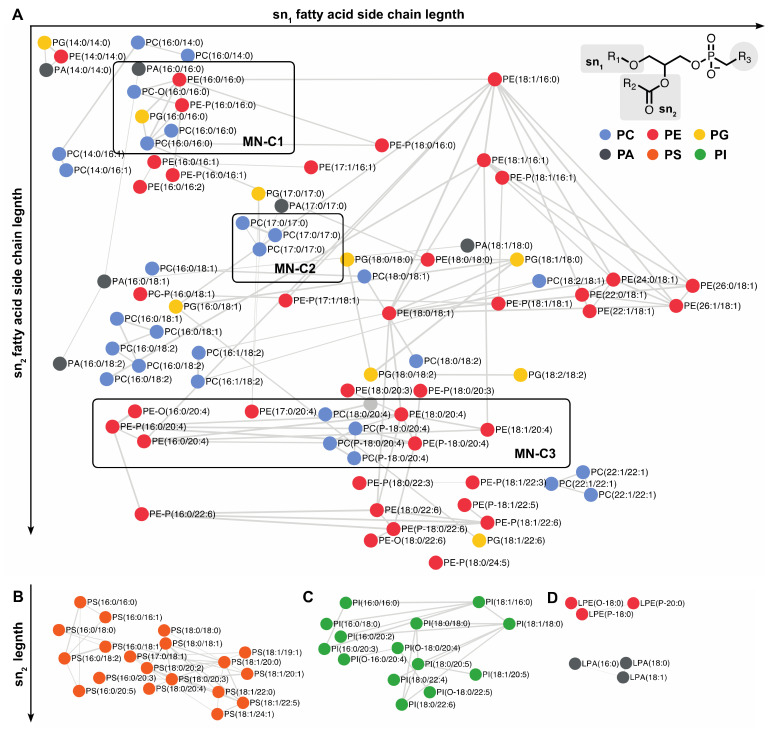
Lipidic molecular network of phospholipids including commercial standards and epithelial corneal cell lipids. The MN between (**A**) PC, PE, PA, and PG; (**B**) PS; (**C**) PI; and (**D**) LP. Networking was based on both fatty acyl side chains and polar head groups. The MN was organized along two orthogonal axes: abscissa and ordinate correspond to the sn_1_ and sn_2_ fatty acids, respectively. See the text for the explanation of the MN–C1, MN–C2, and MN–C2.

**Figure 5:**
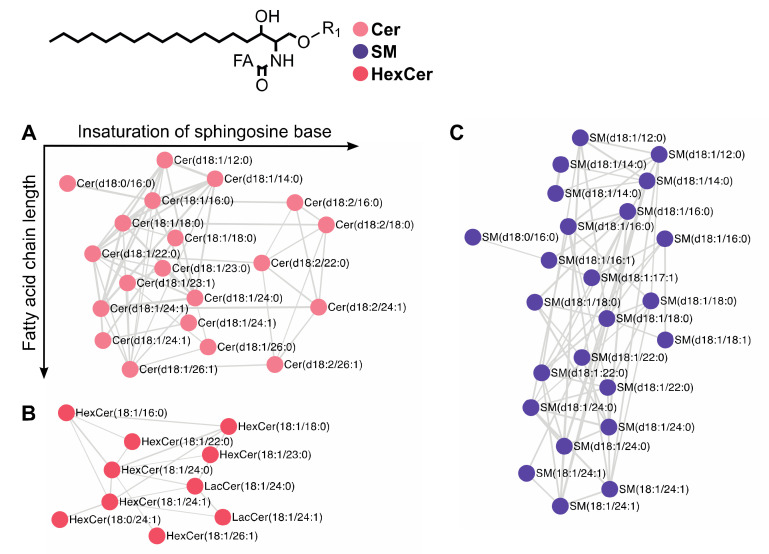
Lipidic molecular network of sphingolipids including commercial standards and epithelial corneal cell lipids. MN of (**A**) ceramide, (**B**) sphingomyelin, and (**C**) hexosylceramide. The MN was organized along two orthogonal axes: abscissa and ordinate corresponding to insaturation of the sphinganine base and fatty acid side chain length, respectively.

In our case, MNs were used as part of a study aimed at assessing the impact on the HCE cell lipidome exposed to HO and were implemented as described hereafter. Tandem mass spectrometry in the DDA mode (see Section 3.3) was used to acquired MS and MS/MS data for 65 commercial standard lipids and for the whole lipids contained in HCE cell extracts. Next to a preprocessing step performed with MzMine 2 (see Section 3.4), preprocessed data were subsequently used to build MNs through the GNPS platform. The MNs thus included three types of nodes corresponding first, to commercial standard lipids, second, to lipids available both as commercial references and identified in HCE cell extracts, and, finally, to lipids only detected in HCE cell extracts (Figure 4 and Figure 5, Table S3).

In such a MN, commercial lipid standards of known structure were thus used to anchor the molecular network within which lipids from HCE cell extracts were clustered according to the similarities of structures that they shared with reference lipids. Thanks to the standard lipids, the key parameters were optimized to provide a reliable and relevant MN. The nodes of the network, corresponding to the MS/MS spectra, were only linked to others if they displayed a common fragmentation pattern, *i.e.*, a minimum number of six identical product ions and/or neutral losses. Moreover, the similarity score between a pair of MS/MS spectra, also called “cosine score” (cos) had to be greater than 0.6. Values selected for the aforementioned parameters were widely used for molecular networking [21].

Figure 4 corresponds to the network of phospholipids; it includes PC, PE, PA, and PG. It was mainly built from the fatty acyl chains located at the sn_1_ and sn_2_ positions, and, to a less extent, to the polar head group. For instance, various phospholipid species (*i.e.*, PC, PE, PC-P, PE-P, PG, PA) containing palmitate in the sn_1_ and sn_2_ positions were clustered (cluster MN-C1 in Figure 4). The PC (16:0/18:1) and PC (18:0/18:1) were also clustered, as they both contained oleate in the sn_2_ position and a phosphocholine polar head group (Figure 4). Similarly, PC (18:0/18:1) and PC (18:0/18:2) were clustered, as they contained stearate in the sn_1_ position (Figure 4). In some cases, depending on the precursor ion intensity, the MN could also include several adducts corresponding to only one PC. This is especially the case for the lipid standard PC (17:0/17:0) observed as [M−CH_3_]^−^, [M+HCOO]^−^ and [M+CH_3_COO]^−^ ion species (cluster MN–C2 in Figure 4). Phospholipids containing ether (PC–O and PE–O) or vinyl ether (PC–P and PE–P) bonds in the sn_1_ position are displayed on the same MN as the diacylphospholipids. For instance, PE–P (16:0/16:1) included in MN–C1 was connected to PE–P (16:0/16:0), as they both contained a phosphoethanolamine head group and a sn_1_ palmitoyl moiety. The PE–P (16:0/16:0) was also connected to PC–O (16:0/16:0) and PE (16:0/16:0), as they all contained the same two palmitoyl moieties. In contrast, PE–P (16:0/16:1) was not connected to PE (16:0/16:1), indeed, their MS/MS spectra displayed only four common product ions, this being insufficient to connect them in the MN. To improve data visualization, the MN was organized under two orthogonal axes; the abscissa and the ordinate corresponding, respectively, to the sn_1_ and sn_2_ fatty acyl chains of glycerophospholipids. For instance, lipids containing an arachidonate side chain in the sn_2_ position were displayed on the same line (cluster MN–C3 in Figure 4).

It must be emphasized that, in contrast to other phospholipids, PS and PI clustering was mainly based on their polar head group because of abundant characteristic fragment ions (Figure 4B,C). The [M−H]^−^ precursor ions of PS were dissociated by the loss of the serine moiety leading to an intense [M−H−87.0326]^−^ product ion [35] (Figure S2). The MS/MS spectra of the deprotonated PI displayed an inositol phosphate fragment at *m/z* 259.0225 and two product ions corresponding to the consecutive loss of one (*m/z* 241.0119) and two (*m/z* 223.0013) water molecules (Figure S3).

**Figure 6:**
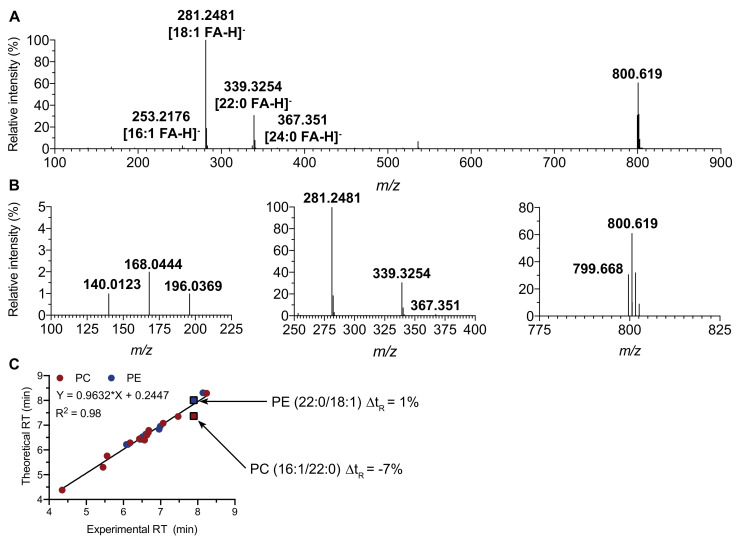
Retention time prediction use to discriminate two isobaric PE and PC species. (**A**) MS/MS spectrum of PE (40:1) [M−H]^−^ ion (*m/z* 800,619). (**B**) Low, middle, and high mass region of the spectrum A. (**C**) Theoretical t_R_ plotted against experimental t_R_ for standard PC and PE species (circles) and for PE (22:0/18:1) and PC (16:1/22:0) (squares).

The networks connecting sphingolipids were all organized by subclasses (Figure 5). Ceramides (Figure 5A) were thus clustered separately from sphingomyelins (Figure 5B) and hexosylceramides (Figure 5C). Furthermore, networks of each sphingolipid subclasses displayed clusters depending on sphinganine (d18:0), sphingosine (sphing-4-enine, d18:1) or a sphingadienine (sphing-4,14-dienine, d18:2) moiety. In the ceramide subclass, Cer (d18:1/26:0) was connected to Cer (d18:1/26:1), as they both contained a sphingosine (d18:1) moiety. Clustering also depended on the nature of fatty acyl chain as exemplified by Cer (d18:0/16:0) which was connected to Cer (d18:1/16:0), as they both contained a palmitoyl moiety but displayed no connection with Cer (d18:1/18:0). To simplify the data visualization in Figure 6, the MN was organized on two orthogonal axes: the abscissa and the ordinate correspond to the number of insaturation of the sphinganine base moiety and to the fatty acyl chain length, respectively.

### 2.6. Use of Retention Time Prediction for Lipid Annotation

Applied to HCE cell lipidome, the MN approach was helpful to annotate more than 150 phospholipid and sphingolipid species (Table S3). Annotation was based on tandem mass spectrometry and confirmed by retention time with a maximum tolerance of 5% between theoretical and experimental t_R_ values. In some cases, a co-elution and a co-selection of precursor ions in MS/MS was encountered leading to difficult mass spectra interpretation which did not readily permit to decide between two different lipid structures. In such a case, the t_R_ prediction made it possible to annotate unequivocal lipid species. 

For instance, the MS/MS spectrum of the ion at *m/z* 800.619 displayed the characteristic fragment ions at *m/z* 140.0123 and 196.0369 of a phosphoethanolamine headgroup suggesting a PE (40:1) structure. The spectrum also exhibited intense peaks corresponding to oleate (*m/z* 281.2480) and behenate (*m/z* 339.3254) but also two small peaks at *m/z* 253.2175 and 367.3488 indicative of palmitoleate and lignocerate, respectively (Figure 6A,B). The selected precursor ion thus corresponded to a mixture of an abundant PE (22:0/18:1) and a less abundant PE (24:0/16:1). However, the presence of an ion at *m/z* 168.0444 could correspond to the headgroup of an isobaric [M−CH_3_]^−^ precursor ion from PC (16:1/22:0). Thanks to the t_R_ prediction, PC (16:1/22:0) was excluded as the difference between experimental and theoretical t_R_ was 7% (Figure 6C). The origin of the phosphocholine ion at *m/z* 168.0444 was explained by the co-elution and co-selection by the Q1 quadrupole of the demethylated SM (d42:1) at *m/z* 799.668, a very scarce species in the precursor ion beam. This annotation was confirmed by a tiny difference of experimental and theoretical t_R_ (0.3%). 

### 2.7. Use of Existing Lipid Library Database

Although the proposed annotation procedure is performed through an individual inspection of each MS/MS spectrum, an automatization of the annotation may, at least in part, be considered using currently available lipid library especially LipidBlast or Lipidex [41,42,43].

For example, using LipidBlast, we performed annotation of the commercial standard lipids available in this study. Among the 65 lipids species studied, 50 were successfully annotated using LipidBlast and one misannotation was reported (Figure S4). None of the six SM species were annotated as SM subclass is not supported by the LipidBlast library using negative ion mode LC-ESI-MS/MS data. Moreover, among the 13 PC species included in the commercial standard lipid mixture, three PC failed to be annotated using LipidBlast.

In the frame of a lipidomic analysis, MN in combination with lipid databases may be regarded as a valuable and saving time approach giving a strong insight in the structural elucidation of lipid. Nevertheless, we believe that it remains important to use at least several known standard lipids to perform annotation of unknown lipid species with a high degree of confidence.

### 2.8. Effect of HO on HCE Cells

The lipid annotation through molecular networking and the retention time prediction approach proposed in this study was applied to assess lipid perturbations in human corneal epithelial cells exposed to hyperosmolarity (HO)—an *in vitro* model of dry eye disease [27,28]. Dry eye disease is a chronic inflammatory pathology of the ocular surface. It is characterized by alteration of tear film, HO, cell damage, and inflammation of the ocular surface, all contributing to a vicious circle [30,31,44,45]. Therapeutic strategies targeting inflammatory processes, such as cyclosporine or other anti-inflammatory agents, have been proposed to break this deleterious cycle [30,46]. To better understand the mechanism underlying DED and to find new marker of this pathology, especially to improve patient monitoring and to develop new targeted treatments, further investigations are still needed.

Hyperosmolarity is a key feature of DED [31]. Indeed, it induces significant stress targeting the corneal cell membrane [28,47,48]. Studying the modification of lipid homeostasis related to HO is relevant as this stressor induces the disruption of processes closely associated to cell membranes. Indeed, HO activates pro-inflammatory and pro-apoptotic processes, initiated at the cell membrane level. Hyperosmolarity stimulates downstream signaling pathways mediated by multiple membrane-bound proteins and enzymes [27,49,50,51]. The interest to study cell lipid disruption is also reinforced as HO favors ROS production which, in turn, targets lipids through peroxidation [52,53,54].

**Figure 7:**
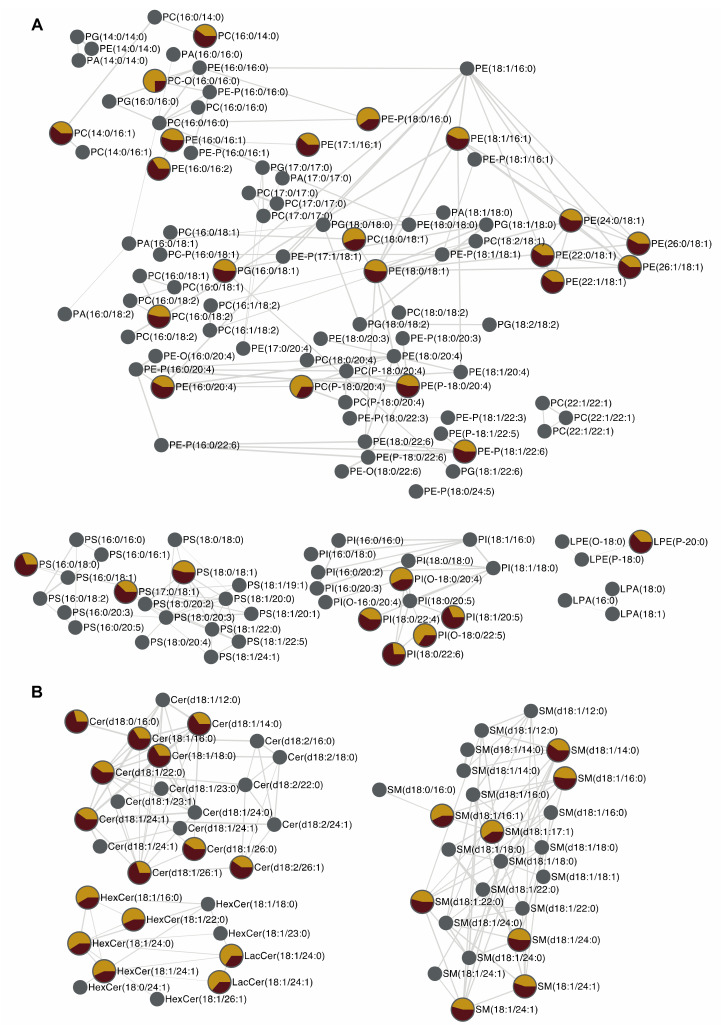
MN of lipids up- or downregulated in human corneal epithelial cells exposed to HO. MN displays (**A**) phospholipids and (**B**) sphingolipids species up- or downregulated in human corneal epithelial cells exposed to HO. The pie chart represents mean ion intensity of a lipid molecule from the control cell (yellow) and HO exposed cell (red) lipidomes. Of note, networking, based on the structural characteristics of lipid species, does not provide information regarding biosynthesis and modeling/remodeling pathways of the lipids found within HCE cells, especially under the effect of hyperosmolarity.

Lipidomic analysis associated to molecular networking and t_R_ calculation led to the annotation of 150 lipid species and revealed that among them, 54 phospholipids and sphingolipids were significantly up- or downregulated in human corneal epithelial cells exposed to HO (Figure 7).

Regarding phospholipids, an increase of the cell concentration was observed for 4 PC, 10 PE, 3 PS, and 3 PI species under HO exposure. These phospholipids mainly contained oleate (18:1) located in the sn_2_ position. On the contrary, abundances of six ether-phospholipid species were strikingly decreased. Thanks to MN, they were identified as 2 PC-P, 2 PI-O, and 2 PE-P species containing polyunsaturated fatty acids at position sn_2_, especially arachidonic (20:4) and docosahexaenoic (22:6) acids (Figure 7). Ether phospholipids are known to be pools of FA (22:6) and FA (20:4) [55]. The FA (20:4) is the preferential substrate of cyclooxygenase (COX) and lipoxygenase (LOX), enzymes leading to pro-inflammatory eicosanoids. Therefore, our results suggest that HO, a key feature in DED, may favor the release of arachidonic acid from ether phospholipids to promote inflammatory process. Ether phospholipid, especially PE-P and PC-P species, are also known to be targets of oxidative stress through vinyl ether bonds. Beside inflammation, oxidative stress is also a key feature of DED pathophysiology [44,56]. Therefore, the significant decrease in PC-P and PE-P species observed in HCE cells under HO exposure may proceed through oxidation of these lipids which is known to generate toxic malondialdehyde (MDA) and 4-hydroxynonenal (4-HNE). Of note, MDA and 4-HNE release have previously been described in DED *in vitro* models as well as in conjunctival imprints of DED suffering patients [53,57].

Regarding sphingolipids, an increase of ceramide abundance was observed in HCE cells submitted to HO (Figure 7). Ceramides are bioactive lipid species known to promote inflammation through IL-1β release and to induce apoptosis through caspase 3 activation [7,58]. Dysregulation of ceramide metabolism is involved in many inflammatory diseases such as atherosclerosis, inflammatory bowel disease or multiple sclerosis [2,3]. In the frame of DED, apoptosis and inflammatory processes induced by ceramides may thus be considered as important mediators of the deleterious effects of HO in accordance with previous published data [47,59]. Thanks to MN, Cer (d18:0/16:0) and Cer (d18:1/16:0) were successfully identified. Under HO exposure, these two lipids species are increased in HCE cells. Because Cer (d18:0/16:0) and Cer (d18:1/16:0) are respectively substrate and a product of dehydroceramide desaturase—a key enzyme in *de novo* synthesis of ceramide—this result suggests that HO promotes *de novo* synthesis of ceramides making this ceramide biosynthetic pathway a putative therapeutic target in the frame of DED.

## 3. Materials and Methods 

### 3.1. Chemicals and Reagents

Chloroform (Carlo Erba Reactifs SDS, Val-de-Reuil, France), acetonitrile, methanol, isopropanol, water of LC-MS grade (J.T. Baker, Phillipsburg, NJ, USA) and 3,5-di-*tert*-4-butylhydroxytoluene (Sigma–Aldrich, Saint-Quentin Fallavier, France) were used to perform cell lipid extraction and to prepare mobile phase for liquid chromatography. All commercial lipid standards were purchased from Avanti Polar Lipids, Inc. (Alabaster, AL, USA) and are listed in Table S1 of the Supplementary Materials.

### 3.2. Sample Preparation

The HCE cells were exposed to HO (500 mOsM) for 24 h and then were washed with Dulbecco’s Phosphate-Buffered Saline (DPBS). Cells were harvested using trypsin-EDTA 0.05%, washed with DPBS, centrifuged at 2000 rpm for 10 min. Dry cell pellets were adjusted to 3 million cells and stored at −80 °C until analysis. After thawing, the cell pellets were resuspended in ultra-pure water (1 mL) and were sonicated for 5 min. Lipids were extracted using a chloroform/methanol/water (5:5:2, *v/v/v*) mixture containing 3,5-di-*tert*-4-butylhydroxytoluene 0.01% (*w/v*) as an antioxidant agent. Samples were subsequently centrifuged at 3000 rpm for 10 min, organic phases were collected, and solvents were evaporated under reduced pressure at 45 °C. Dry residues were dissolved in a 100 µL mixture containing acetonitrile/isopropanol/chloroform/water (35:35:20:10, *v/v/v/v*) before injection into the UHPLC-MS system.

### 3.3. Data-Dependent LC-ESI-HRMS/MS Analysis 

Liquid chromatography-negative electrospray ionization mass spectrometry analysis of lipid extracts was performed on a UHPLC system (Waters^®^, Manchester, UK) combined with a Synapt^®^G2 High Definition MS™ (Manchester, UK) (Q-TOF) mass spectrometer (Waters^®^). Chromatographic separation was achieved on an Acquity^®^ (Manchester, UK) CSH C18 column (100 × 2.1 mm; 1.7 µm). Lipids were eluted using a binary gradient system consisting in 10 mM ammonium acetate in acetonitrile/water mixture (40:60, *v/v*) as solvent A and 10 mM ammonium acetate in acetonitrile/isopropanol mixture (10:90, *v/v*) as solvent B. The eluent increased from 40% B to 100% B in 10 min, was held at 100% B for 2 min before returning to 40% B. The flow rate was kept at 0.4 mL.min^−1^. The column oven was set at 50 °C and the injection volume was 5 µL. The source parameters were as follows: capillary voltage 2400 V, cone voltage 45 V, source temperature 120 °C, desolvation temperature 550 °C, cone gas flow 20 L h^−1^, and desolvation gas flow 1000 L h^−1^. Leucine enkephalin (2 ng mL^−1^) was used as an external reference compound (Lock-Spray™, Manchester, UK) for mass correction. In a data-dependent acquisition mode (DDA), MS full scans were followed by MS/MS scans performed on the five most intense ions above an absolute threshold of 1000 counts. Selected parent ions were fragmented at collision energy ramp 20–40 eV and a selection window size of 1.0 Th. Scan durations for both MS and MS/MS were 0.2 s. In the full scan mode, the data were acquired between *m/z* 50 and 1200 using a resolution of 20,000 FWHM at *m/z* 500. Data acquisition was managed using Waters MassLynx™ software (version 4.1; Waters MS Technologies, Manchester, UK). A mixture of 65 standard lipids belonging to 9 of the main lipid classes (*i.e.*, phosphatidic acid (PA), phosphatidylethanolamine (PE), phosphatidylserine (PS), phosphatidylcholine (PC), phosphatidylglycerol (PG), ceramide (Cer), sphingomyelin (SM), HexosylCeramide (HexCer)) at a final individual concentration of 1 µM was also periodically injected throughout the analytical batch.

### 3.4. Data-Preprocessing Parameters

Raw data files were converted into universal open source mzXML file with MSConvert 3.0 and were then processed using MZmine 2.51 software. The MS and MS/MS spectra were extracted using MZmine 2.51 with a mass detection noise level set at 2E2 and 0E0, respectively. Chromatograms were then built with the ADAP algorithms [60] using a minimum group size of 5 scans, a group intensity threshold of 5000, and an *m/z* tolerance of 0.005 Da (about 10 ppm). The ADAP wavelets chromatogram deconvolution algorithm was used with the following settings: signal-to-noise ratio = 10, coefficient/area ratio = 50, peak duration range = 0.05−0.4 min, retention time wavelet range = 0.02–0.1, *m/z* range for MS/MS scan pairing of 0.01, and t_R_ range for MS/MS scan pairing of 0.15 min. Chromatograms were de-isotoped using the isotopic peaks grouper algorithm with a *m/z* tolerance set at 0.005 (*m/z* < 500) and 10 ppm (*m/z >* 500), and a t_R_ tolerance of 0.1 min. Peak alignment was performed using the join aligner method using the following parameters: *m/z* tolerance at 0.005 (*m/z* < 500) and 10 ppm (*m/z >* 500) and an absolute t_R_ tolerance 0.15 min. Each MS/MS scans were associated with the corresponding MS scans using a t_R_ tolerance of 0.1 min and a *m/z* tolerance of 0.005 (*m/z* < 500) and 10 ppm (*m/z >* 500). The peak list was finally gap-filled using the so-called module “same RT and *m/z* range gap filler” with *m/z* tolerance 0.005 (*m/z* < 500) and 5 ppm (*m/z >* 500).

### 3.5. Molecular Network Analysis 

The MNs were created using the feature based molecular networking workflow of the Global Natural Products Social (GNPS) platform [61]. The following settings were used to build the network: minimum pairs Cos > 0.60, parent ion mass tolerance = 0.02 Da, fragment ion mass tolerance = 0.02, network topK < 100, minimum matched peaks = 6, and minimum cluster size = 2. The library spectra inquiries were performed using the same parameter values as those define for the network building. The MNs were finally visualized and annotated using Cytoscape 3.4.0 software (San Diego, California, USA) [62].

### 3.6. Lipid Structure Assignment

The structural annotation of unknown lipid species was based on the MNs generated on the GNPS platform using MS and MS/MS data as follow: (*i*) nodes associated to lipid standards were indexed using MzMine 2 thanks to MS, MS/MS data, and t_R_ value, (*ii*) nodes associated to unknown lipids were subsequently indexed based on MS data, using online data base LIPIDMAPS and METLIN, and MS/MS data, through manual inspection on MzMine 2, (*iii*) annotation was finally supported by t_R_ values by comparison of experimental t_R_ values to the calculated one using t_R_ prediction models. Based on these three criteria, lipids already annotated were used to create a database valuable for later annotation.

In order to demonstrate the relevance of the MNs in lipid species annotation, MS/MS spectra were individually inspected to select diagnostic product ions essential for annotation and the differences between theoretical and experimental *m/z* values were calculated using Excel software (see Supplementary Materials, compilation of experimental and theoretical *m/z* values of diagnostic product ions for PL and SL).

In accordance with the guidelines provided by the minimum reporting standards of the Metabolomics Standards Initiative [16], Table S3 includes the level of identification for all annotated lipids. Indeed, thanks to accurate *m/z* measurement, the MS/MS data inspection and retention time analysis, lipids annotated in HCE cells were assigned to group 1 or 2. Lipids for which we had the corresponding commercial standards were assigned to group 1. In addition, lipids for which we did not have the corresponding standards, the annotation was performed on the adequacy of *m/z* value, MS/MS data and retention time analysis and were thus assigned to group 2.

### 3.7. Statistical Analysis

A false discovery rate ((FDR)-adjusted *p* < 0.01) controlling procedure was performed to assess the statistical significance of the concentration differences of the identified lipids from cell extracts of HO-treated cells versus control cells. Each experiment was performed independently at least five times. The ANOVA, Dunnett test, and Student *t*-test were performed using GraphPad Prism 8 software (version 8; GraphPad Software, La Jolla, CA, USA) with a risk set at 0.05 (* *p* < 0.05, ** *p* < 0.01, *** *p* < 0.001).

## 4. Conclusions

For the first time, the present study showed that the use of molecular networks makes it possible to facilitate and increase the reliability of lipid annotation in the course of lipidomic analysis. This approach, based on the use of the tandem mass spectrometry DDA mode in negative ionization conditions, allowed characterizing the fatty acyl chains of phospholipids and sphingolipids. In addition, if an ambiguity in the annotation of a lipid persists, the prediction of the retention time makes it possible to remove the latter. This new strategy makes it possible to cover the entire lipidome despite the limited number of standard lipids to which it is possible to have access commercially. The present study was limited to lipid subclasses which had structural characteristics which were clearly depicted by the MN under negative ionization conditions. Nevertheless, through this approach, we were able, in the context of a differential lipidomic analysis of an *in vitro* model of DED (*i.e.*, HCE cells exposed to HO) to annotate many lipids potentially involved in cell death and inflammation. Regarding others lipid subclasses such as glycerolipids, the positive ionization mode appears suitable to highlight their structural characteristics using molecular networks. It is currently in progress and will be the subject of a separate study.

## Supplementary Materials

The following are available online at https://www.mdpi.com/2218-1989/10/6/225/s1, Figure S1: Extracted ion chromatograms of m/z values corresponding to precursor ions of standard phospholipids containing two palmitoyl moieties, Figure S2: MN of PS subclass, Figure S3: MN in PI subclass, Figure S4: Annotation of MS/MS spectra of commercial standard lipids using LipidBlast library, Table S1: Lipid composition of standard mix, Table S2: Repeatability and method precision (within-day, between-day, and intermediate precision), Table S3: Annotation of lipid species by MS/MS experiment. Supplementary Materials include the Excel table used for tR prediction and for diagnostic ion checking.
