# Supplementary material for: Lipid Annotation by Combination of UHPLC-HRMS (MS), Molecular Networking, and Retention Time Prediction: Application to a Lipidomic Study of In Vitro Models of Dry Eye Disease
Source: Metabolites. 2020 May 29;10(6):225. doi: 10.3390/metabo10060225 (PMC7345884; doi:10.3390/metabo10060225)
Supplement: Supplementary file 1 [file metabolites-10-00225-s001.zip › metabolites-785502-supplementary/Supplementary Informations - 200504.pdf]

Supplementary Information

Lipid Annotation by Combination of UHPLC-HRMS(MS), Molecular Networking and Retention Time Prediction: Application to a Lipidomic Study of an In Vitro Models of Dry Eye Disease

Romain Magny[1,2], Anne Regazzetti[2], Karima Kessal[1,3], Gregory Genta-Jouve[2,4], Christophe Baudouin[1,3,5], Stéphane Mélik-Parsadaniantz[1], Françoise Brignole-Baudouin[1,2,3], Olivier Laprévote[2,6] and Nicolas Auzeil[2,*]

[1]Sorbonne Université UM80, INSERM UMR 968, CNRS UMR 7210, Institut de la Vision, IHU ForeSight, Paris, France

[2]UMR CNRS 8038 CiTCoM, Chimie Toxicologie Analytique et Cellulaire, Université de Paris, Faculté de Pharmacie, Paris, France

[3]Centre Hospitalier National d'Ophtalmologie des Quinze-Vingts, IHU ForeSight, Paris, France

[4]Laboratoire Ecologie, Evolution, Interactions des Systèmes Amazoniens (LEEISA), USR 3456, Université De Guyane, CNRS Guyane, French Guiana

[5]Hôpital Ambroise Paré, AP-HP, Université Versailles Saint-Quentin-en-Yvelines, Boulogne-Billancourt, France

[6]Hôpital Européen Georges Pompidou, AP-HP, Service de Biochimie, Paris, France

*Correspondence: nicolas.auzeil@parisdescartes.fr

Table S1A. Lipid composition of standard mix (individual concentration 1 µM): first part

Lipids category	Name	Molecular formula
Ceramides		
Cer(d18:1/12:0)	N-(dodecanoyl)-sphing-4-enine	$C_{30}H_{59}NO_3$
Cer(d18:1/14:0)	N-(tetradecanoyl)-sphing-4-enine	$C_{32}H_{63}NO_3$
Cer(d18:1/16:0)	N-(hexadecanoyl)-sphing-4-enine	$C_{34}H_{67}NO_3$
Cer(d18:1/18:0)	N-(octadecanoyl)-sphing-4-enine	$C_{36}H_{71}NO_3$
Cer(d18:1/24:1)	N-(tetracosenoyl)-sphing-4-enine	
Sphingomyelins		
SM(d18:1/16:0)	N-(hexadecanoyl)-sphing-4-enine-1-phosphocholine	$C_{39}H_{79}N_2O_6P$
SM(d18:1/24:1)	N-(tetracosenoyl)-sphing-4-enine-1-phosphocholine	$C_{47}H_{93}N_2O_6P$
SM(d18:1/12:0)	N-(dodecanoyl)-sphing-4-enine-1-phosphocholine	$C_{35}H_{71}N_2O_6P$
SM(d18:1/14:0)	N-(tetradecanoyl)-sphing-4-enine-1-phosphocholine	$C_{35}H_{75}N_2O_6P$
SM(d18:1/18:0)	N-(octadecanoyl)-sphing-4-enine-1-phosphocholine	$C_{41}H_{83}N_2O_6P$
SM(d18:1/24:0)	N-(tetracosanoyl)-sphing-4-enine-1-phosphocholine	$C_{47}H_{95}N_2O_6P$
Galactosylcéramide		
GalCer(d18:1/18:0)	N-(octadecanoyl)-1-β-glucosyl-sphing-4-enine	$C_{42}H_{81}NO_8$
GalCer(d18:1/24:1)	N-(tetracosenoyl)-1-β-glucosyl-sphing-4-enine	$C_{48}H_{91}NO_8$

Table S1B. Lipid composition of standard mix (individual concentration 1 μM): second part

Lipids category	Name	Molecular formula
Phosphatidic acids		
PA(14:0/14:0)	1,2-ditetradecanoyl-*sn*-glycero-3-phosphate	$C_{31}H_{61}O_8P$
PA(16:0/16:0)	1,2-dihexadecanoyl-*sn*-glycero-3-phosphate	$C_{35}H_{69}O_8P$
PA(18:1/18:0)	1-octadecenoyl-2-octadecanoyl-glycero-3-phosphate	$C_{39}H_{75}O_8P$
PA(16:0/18:1)	1-palmitoyl-2-oleoyl-*sn*-glycero-3-phosphate	$C_{37}H_{71}O_8P$
PA(17:0/17:0)	1,2-diheptadecanoyl-*sn*-glycero-3-phosphate	$C_{37}H_{73}O_8P$
PA(16:0/18:2)	1-hexadecanoyl-2-octadecadienoyl-*sn*-glycero-3-phosphate	$C_{37}H_{69}O_8P$
Phosphatidylcholines		
PC(17:0/17:0)	1,2-diheptadecanoyl-*sn*-glycero-3-phosphocholine	$C_{42}H_{84}NO_8P$
PC(18:0/18:2)	1-stearoyl-2-linoleoyl-*sn*-glycero-3-phosphocholine	$C_{44}H_{84}NO_8P$
PC(16:0/18:1)	1-hexadecanoyl-2-octadecenoyl-*sn*-glycero-3-phosphocholine	$C_{42}H_{82}NO_8P$
PC(20:1/20:1)	1,2-dieicosenoyl-*sn*-glycero-3-phosphocholine	$C_{48}H_{92}NO_8P$
PC(16:0/16:0)	1,2-dihexadecanoyl-*sn*-glycero-3-phosphocholine	$C_{40}H_{80}NO_8P$
PC(16:0/18:2)	1-hexadecanoyl-2-octadecadienoyl-*sn*-glycero-3-phosphocholine	$C_{42}H_{80}NO_8P$
PC(18:0/18:1)	1-octadecanoyl-2-octadecenoyl-*sn*-glycero-3-phosphocholine	$C_{44}H_{86}NO_8P$
PC(18:0/20:4)	1-octadecanoyl-2-eicosatetraenoyl-*sn*-glycero-3-phosphocholine	$C_{46}H_{84}NO_8P$
PC(22:1/22:1)	1,2-di-docosenoyl-*sn*-glycero-3-phosphocholine	$C_{52}H_{100}NO_8P$
PC(P-18:0/20:4)	1-octadecenyl-2-eicosatetraenoyl-*sn*-glycero-3-phosphocholine	$C_{46}H_{84}NO_7P$
Phosphatidylethanolamines		
PE(14:0/14:0)	1,2-ditetradecanoyl-*sn*-glycero-3-phosphoethanolamine	$C_{33}H_{66}NO_8P$
PE(16:0/16:0)	1,2-dipalmitoyl-*sn*-glycero-3-phosphoethanolamine	$C_{37}H_{74}NO_8P$
PE(18:0/18:0)	1,2-distearoyl-*sn*-glycero-3-phosphoethanolamine	$C_{41}H_{82}NO_8P$
PE(16:1/16:1)	1,2-dipalmitoleoyl-*sn*-glycero-3-phosphoethanolamine	$C_{37}H_{70}NO_8P$
PE(18:0/18:1)	1-stearoyl-2-oleoyl-*sn*-glycero-3-phosphoethanolamine	$C_{41}H_{80}NO_8P$
PE(18:0/18:2)	1-octadecanoyl-2-octadecadienoyl-*sn*-glycero-3-phosphoethanolamine	$C_{41}H_{78}NO_8P$
PE(18:1/16:1)	1-octadecenoyl-2-hexadecenoyl-glycero-3-phosphoethanolamine	$C_{39}H_{74}NO_8P$
PE(18:1/18:1)	1,2-di-octadecenoyl-*sn*-glycero-3-phosphoethanolamine	$C_{41}H_{78}NO_8P$
PE(16:0/18:1)	1-hexadecanoyl-2-octadecenoyl-*sn*-glycero-3-phosphoethanolamine	$C_{39}H_{76}NO_8P$
PE(17:0/20:4)	1-heptadecanoyl, 2-eicosatetraenoyl-*sn*-glycero-3-phosphoethanolamine	$C_{42}H_{76}NO_8P$
PE(18:0/20:4)	1-octadecanoyl-2-eicosatetraenoyl-*sn*-glycero-3-phosphoethanolamine	$C_{43}H_{78}NO_8P$
PE-P(18:0/20:4)	1-octadecenyl-2-eicosatetraenoyl-*sn*-glycero-3-phosphoethanolamine	$C_{43}H_{78}NO_7P$
PE-P(18:0/22:6)	1-octadecenyl-2-docosahexaenoyl-*sn*-glycero-3-phosphoethanolamine	$C_{45}H_{78}NO_7P$
Phosphatidylglycerols		
PG(14:0/14:0)	1,2-ditetradecanoyl-*sn*-glycero-3-phospho-(1'-*rac*-glycerol)	$C_{34}H_{67}O_{10}P$
PG(16:0/16:0)	1,2-dipalmitoyl-*sn*-glycero-3-phospho-(1'-*rac*-glycerol)	$C_{38}H_{75}O_{10}P$
PG(18:0/18:0)	1,2-distearoyl-*sn*-glycero-3-phospho-(1'-*rac*-glycerol)	$C_{42}H_{83}O_{10}P$
PG(18:2/18:2)	1,2-dilinoleoyl-*sn*-glycero-3-phospho-(1'-*rac*-glycerol)	$C_{42}H_{75}O_{10}P$
PG(17:0/17:0)	1,2-diheptadecanoyl-*sn*-glycero-3-phospho-(1'-*rac*-glycerol)	$C_{40}H_{79}O_{10}P$
PG(16:0/18:1)	1-palmitoyl-2-oleoyl-*sn*-glycero-3-phospho-(1'-*rac*-glycerol)	$C_{40}H_{77}O_{10}P$

PG(12:0/12:0)	1,2-didodecanoyl-*sn*-glycero-3-phospho-(1'-*sn*-glycerol)	$C_{30}H_{59}O_{10}P$
PG(18:1/18:0)	1-octadecenoyl-2-octadecanoyl-glycero-3-phospho-(1'-*sn*-glycerol)	$C_{42}H_{81}O_{10}P$
PG(12:0/13:0)	1-dodecanoyl-2-tridecanoyl-*sn*-glycero-3-phospho-(1'-*rac*-glycerol)	$C_{31}H_{61}O_{10}P$
PG(18:0/18:2)	1-octadecanoyl-2-octadecadienoyl-glycero-3-phospho-(1'-*sn*-glycerol)	$C_{42}H_{79}O_{10}P$
Phosphatidylserines		
PS(14:0/14:0)	1,2-ditetradecanoyl-*sn*-glycero-3-phosphoserine	$C_{34}H_{66}NO_{10}P$
PS(16:0/16:0)	1,2-dipalmitoyl-*sn*-glycero-3-phosphoserine	$C_{38}H_{74}NO_{10}P$
PS(16:0/18:2)	1-palmitoyl-2-linoleoyl-*sn*-glycero-3-phosphoserine	$C_{40}H_{74}NO_{10}P$
PS(17:0/17:0)	1,2-diheptadecanoyl-*sn*-glycero-3-phosphoserine	$C_{40}H_{78}NO_{10}P$
PS(12:0/13:0)	1-dodecanoyl-2-tridecanoyl-*sn*-glycero-3-phosphoserine	$C_{31}H_{60}NO_{10}P$
PS(18:0/18:1)	1-octadecanoyl-2-octadecenoyl-*sn*-glycero-3-phosphoserine	$C_{42}H_{80}NO_{10}P$
Phosphatidylinositol		
PI(18:0/18:0)	1,2-dioctadecanoyl-*sn*-glycero-3-phospho-(1'-*myo*-inositol)	$C_{45}H_{87}O_{13}P$
PI(18:0/20:4)	1-octadecanoyl-2-eicosatetraenoyl-*sn*-glycero-3-phospho-(1'-*myo*-inositol)	$C_{47}H_{83}O_{13}P$
PI(16:0/16:0)	1,2-dihexadecanoyl-*sn*-glycero-3-phospho-(1'-myo-inositol)	$C_{41}H_{79}O_{13}P$

Table S2. Repeatability and method precision (within-day, between-day and intermediate precision) according to Kouassi *et al.*, *Anal Chem.*, 2017

	m/z						t_R			
**Standard lipids**	**Theoretical *m/z***	**Mean *m/z* (*n* = 18)**	**Mean Δ ppm n=18**	**ppm Within-Day**	**ppm Between-Day**	**ppm Intermediate Precision**	**Mean t_R**	**CV% Within-Day**	**CV % Between-Day**	**CV% Intermediate Precision**
Cer d18:1/12:0)	480,4429	480,4415	3,1	1,1	0,8	1,4	5,36	0,2	0,2	0,3
Cer(d18:1/14:0)	508,4735	508,4736	0,9	1,2	0,4	1,3	5,99	0,1	0,4	0,4
Cer(d18:1/16:0)	536,5048	536,5042	1,4	1,1	0,4	1,2	6,56	0,1	0,4	0,4
Cer(d18:1/18:0)	564,5356	564,5352	1,1	1,4	0,4	1,4	7,07	0,2	0,4	0,4
Cer(d18:1/24:1)	646,6143	646,6125	2,9	1,4	0,7	1,6	7,88	0,2	0,2	0,3
GalCer(d18:1/18:0)	726,5888	726,5868	2,9	1,5	0,7	1,6	6,61	0,2	0,4	0,5
GalCer(d18:1/24:1)	808,6664	808,6653	1,6	1,2	0,1	1,2	7,48	0,1	0,3	0,3
PC(16:0/16:0)	718,5392	718,5374	2,9	2,0	1,0	2,3	6,42	0,2	0,4	0,5
PC(16:0/18:1)	744,5548	744,5529	2,6	1,6	1,3	2,0	6,48	0,2	0,4	0,4
PC(16:0/18:2)	742,5392	742,5372	2,8	1,3	1,1	1,7	6,09	0,0	0,5	0,5
PC(17:0/17:0)	746,5705	746,5696	1,9	2,1	0,5	2,1	6,94	0,1	0,0	0,1
PC(18:0/18:1)	772,584	772,5841	0,9	1,1	0,3	1,1	6,98	0,2	0,4	0,4
PC(18:0/18:2)	770,5705	770,5687	2,7	1,7	0,9	1,9	6,61	0,2	0,4	0,5
PC(18:0/20:4)	794,5716	794,5735	2,7	1,6	0,6	1,7	6,62	0,0	0,6	0,6
PC(20:1/20:1)	826,6344	826,6360	2,9	1,4	0,5	1,5	7,42	0,2	0,3	0,3
PC(22:1/22:1)	942,7161	942,7147	1,7	1,3	0,8	1,5	8,06	0,2	0,3	0,3
PC(P-18:0/20:4)	778,5756	778,5738	2,3	1,2	0,3	1,2	6,78	0,2	0,5	0,5

	m/z						**t_R**			
**Standard lipids**	**Theoretical *m/z***	**Mean *m/z* (*n* = 18)**	**Mean Δ ppm n=18**	**ppm Within-Day**	**ppm Between-Day**	**ppm Intermediate Precision**	**Mean t_R**	**CV% Within-Day**	**CV % Between-Day**	**CV% Intermediate Precision**
PE(14:0/14:0)	634,4442	634,4442	1,0	1,1	0,6	1,3	5,39	0,2	0,5	0,6
PE(16:0/16:0)	690,5071	690,5057	2,0	1,0	0,4	1,0	6,55	0,2	0,4	0,5
PE(18:0/18:0)	746,5695	746,5681	2,4	2,0	0,9	2,2	7,45	0,1	0,3	0,4
PE(18:1/16:1)	714.507	714,5087	2,4	2,2	0,5	2,2	6,18	0,6	1,0	1,2
PE(16:1/16:1)	686,4766	686,4744	3,3	1,2	0,4	1,3	5,57	0,1	0,5	0,6
PE(18:1/18:1)	742,5401	742,5375	4,2	2,2	0,7	2,3	7,03	0,3	0,4	0,5
PE(17:0/20:4)	752,5235	752,5219	2,8	2,2	1,5	2,7	6,40	0,2	0,4	0,4
PE(18:0/18:1)	744,5549	744,5532	2,3	1,6	0,5	1,7	7,06	0,2	0,3	0,4
PE(18:0/18:2)	742,5392	742,5375	2,4	1,1	0,9	1,4	6,72	0,2	0,5	0,5
PE(16:0/18:1)	716.523	716,5244	1,3	0,8	0,1	0,9	**6,63**	0,1	0,4	0,4
PE(18:0/20:4)	766,5392	766,5379	2,0	1,8	0,6	1,9	6,66	0,2	0,4	0,4
PE(P-18:0/20:4)	750,5443	750,5402	5,6	3,3	1,9	3,8	6,90	0,2	0,4	0,5
PE(P-18:0/22:6)	774,5443	774,5402	5,3	2,4	0,9	2,6	6,78	0,2	0,5	0,5
PG(12:0/12:0)	609,3764	609,3763	1,2	1,4	0,6	1,5	2,97	0,4	1,2	1,2
PG(14:0/14:0)	665,4375	665,4384	1,7	1,5	0,4	1,6	4,31	0,2	0,9	0,9
PG(16:0/16:0)	721,5014	721,5008	1,2	1,1	0,6	1,2	5,50	0,2	0,6	0,6
PG(18:0/18:0)	777,5647	777,5632	2,0	1,5	0,5	1,6	6,47	0,3	0,6	0,6
PG(18:1/18:0)	775,5486	775,5477	1,7	1,5	0,4	1,6	6,08	0,2	0,6	0,7
PG(12:0/13:0)	623,3929	623,3917	1,9	1,2	0,4	1,3	3,32	0,4	1,6	1,6

	m/z						t_R			
**Standard lipids**	**Theoretical *m/z***	**Mean *m/z* (*n* = 18)**	**Mean Δ ppm n=18**	**ppm Within-Day**	**ppm Between-Day**	**ppm Intermediate Precision**	**Mean t_R**	**CV% Within-Day**	**CV % Between-Day**	**CV% Intermediate Precision**
PG(16:0/18:1)	747,5181	747,5156	3,4	0,9	0,3	0,9	5,59	0,2	0,7	0,7
PG(17:0/17:0)	749,5338	749,5320	2,5	1,1	0,6	1,3	6,02	0,1	0,6	0,7
PG(18:0/18:2)	773,5338	773,5322	2,1	1,2	0,2	1,2	5,71	0,2	0,6	0,7
PG(18:2/18:2)	769,5025	769,5005	2,6	1,4	0,2	1,4	4,83	0,2	0,8	0,8
PI(16:0/16:0)	809,5172	809,5174	1,4	1,8	0,7	2,0	5,38	0,3	0,6	0,6
PI(18:0/18:0)	865,5791	865,5798	1,3	1,3	0,5	1,4	6,39	0,2	0,5	0,6
PI(18:0/20:4)	885,5482	885,5490	1,3	1,7	0,5	1,8	5,56	0,2	0,7	0,7
PS(18:0/18:1)	788,5441	788,5428	1,9	1,5	0,5	1,6	6,12	0,3	0,7	0,7
PS(14:0/14:0)	679,4322	679,433	1,0	1,3	0,3	1,3	4,4	0,9	1,5	1,7
PS(17:0/17:0)	762,5264	762,5274	1,3	1,7	0,5	1,8	6,14	0,2	0,7	0,7
PS(12:0/13:0)	636,3882	636,3866	2,8	2,6	0,6	2,7	3,21	0,3	1,1	1,2
PS(16:0/16:0)	734,4977	734,4952	3,4	1,4	0,6	1,5	5,52	0,2	0,6	0,6
PS(16:0/18:2)	758,4977	758,4954	3,1	1,1	0,5	1,2	5,16	0,4	0,7	0,8
SM(d18:1/12:0)	631,4803	631,4807	1,2	1,4	0,4	1,4	4,52	0,3	0,6	0,7
SM(d18:1/14:0)	659,5113	659,5116	1,3	1,7	0,6	1,8	5,22	0,2	0,6	0,6
SM(d18:1/16:0)	687,5446	687,5424	3,1	1,1	0,7	1,3	5,86	0,2	0,5	0,5
SM(d18:1/18:0)	715,5747	715,5737	1,6	1,4	0,2	1,4	6,43	0,2	0,4	0,5
SM(d18:1/24:0)	799,6698	799,6675	2,9	1,3	0,9	1,6	7,78	0,3	0,3	0,4
SM(d18:1/24:1)	797,6544	797,6523	2,7	1,1	0,8	1,4	7,35	0,2	0,3	0,4

	m/z						t_R			
**Standard lipids**	**Theoretical *m/z***	**Mean *m/z* (*n* = 18)**	**Mean Δ ppm n=18**	**ppm Within-Day**	**ppm Between-Day**	**ppm Intermediate Precision**	**Mean t_R**	**CV% Within-Day**	**CV % Between-Day**	**CV% Intermediate Precision**
PA(12:0/13:0)	549,3531	549,3510	3,4	1,4	0,6	1,5	3,54	0,9	1,5	1,7
PA(16:0/18:1)	673,4787	673,4790	1,0	1,1	0,6	1,3	5,87	0,2	0,8	0,8
PA(18:0/18:0)	703,5258	703,5270	2,1	1,2	0,2	1,2	6,85	0,4	0,7	0,8
PA(16:0/16:0)	647,463	647,4640	1,9	1,2	0,4	1,3	5,91	0,2	0,3	0,4
PA(14:0/14:0)	591,4002	591,4013	1,9	1,5	0,5	1,6	4,69	0,2	0,7	0,7
PA(17:0/17:0)	675,4944	675,4930	1,9	1,5	0,5	1,6	6,43	0,3	0,7	0,7
PA(18:1/18:0)	701,5101	701,5120	2,8	2,6	0,6	2,7	6,17	0,2	0,5	0,6

Table S3A. Annotation of lipid species by MS/MS experiment of phospholipid fragmentation. Fragmentations were acquired from the precursor ion. Annotation of product ions according to Hsu *et al.*, *J. Chromatogr. B*, 2009. Thanks to accurate *m/z* measurement, MS/MS data inspection and retention time analysis, lipids annotated in HCE cells were assigned to group 1 or 2 according to the guidelines provided by the minimum reporting standards of the Metabolomics Standards Initiative. Annotated lipids labelled with * are indicative of standard lipids whereas those labelled with ** are indicative of standard lipids also found in HCE cells. CSL Mix = Commercial Standard Lipid Mixture.

Name	t_R min (Δ%)	Adduct	Precursor ion *m/z* (Δ ppm)	Polar head group *m/z* (Δ ppm)	$[R_1CH_2\text{-}COO]^-$ *m/z* (Δ ppm)	$[R_2CH_2\text{-}COO]^-$ *m/z* (Δ ppm)	$[M\text{-}H\text{-}R_1'\text{-}CH{=}C{=}O]^-$ *m/z* (Δ ppm)	$[M\text{-}H\text{-}R_2'\text{-}CH{=}C{=}O]^-$ *m/z* (Δ ppm)	Present in CSL Mix	Present in Cells	Group
PA(14:0/14:0) *	4.69 (3)	$[M\text{-}H]^-$	591.4013 (2)	152.9961 (2)	227.2005 (5)	227.2005 (5)	363.1894 (3)	363.1894 (3)	Yes	No	1
PA(16:0/16:0) **	5.91 (3)	$[M\text{-}H]^-$	647.464 (2)	152.9977 (13)	255.2332 (1)	255.2332 (1)	391.2246 (7)	391.2246 (7)	Yes	Yes	1
PA(16:0/18:1) **	5.87 (2)	$[M\text{-}H]^-$	673.479 (1)	152.9963 (4)	255.233 (0)	281.2488 (0)	417.2394 (5)	391.2276 (15)	Yes	Yes	1
PA(16:0/18:2) *	5.29 (6)	$[M\text{-}H]^-$	671.461 (2)	152.9935 (15)	255.226 (27)	279.229 (14)	415.2408 (46)	391.2244 (7)	Yes	No	1
PA(17:0/17:0) *	6.43 (1)	$[M\text{-}H]^-$	675.4944 (0)	152.9944 (9)	269.2483 (1)	269.2483 (1)	405.2406 (8)	405.2406 (8)	Yes	No	1
PC-O(16:0/14:0)	6,25 (1)	$[M\text{-}CH_3]^-$	676.525 (7)	168.0432 (7)	/	227.2007 (4)	/	466.3166 (29)	No	Yes	2
PC-O(16:0/16:0)	6,72 (0)	$[M\text{-}CH_3]^-$	704.551 (14)	168.0444 (14)	/	255.232 (4)	/	466.3292 (2)	No	Yes	2
PC-O(16:0/16:0)	6,78 (1)	$[M\text{+}OAc]^-$	778.5921 (8)	168.0432 (8)	/	255.2316 (5)	/	466.3211 (19)	No	Yes	2
PC-O(18:0/16:0)	7,21 (1)	$[M\text{+}Formiate]^-$	792.614 (0)	168.0431 (0)	/	255.2314 (6)	/	494.3611 (1)	No	Yes	2
PC-O(18:0/16:0)	7,21 (1)	$[M\text{-}CH_3]^-$	732.589 (5)	168.043 (5)	/	255.2319 (4)	/	494.358 (7)	No	Yes	2
PC-P(16:0/16:0)	6,73 (0)	[M+OAc]-	776.581 (2)	168.043 (2)	/	255.2332 (1)	/	464.3148 (1)	No	Yes	2
PC-P(16:0/16:0)	6,73 (0)	$[M\text{-}CH_3]^-$	702.54 (7)	168.039 (7)	/	255.2321 (3)	/	464.3207 (14)	No	Yes	2
PC-P(16:1/22:2)	7,47 (5)	$[M\text{-}CH_3]^-$	780.591 (2)	168.043 (2)	/	335.295 (2)	/	462.3002 (3)	No	Yes	2
PC-P(16:1/22:3)	7,18 (6)	$[M\text{-}CH_3]^-$	778.5702 (8)	168.0432 (8)	/	333.2781 (6)	/	462.3033 (10)	No	Yes	2

Name	t_R min (Δ%)	Adduct	Precursor ion *m/z* (Δ ppm)	Polar head group *m/z* (Δ ppm)	$[R_1CH_2\text{-}COO]^-$ *m/z* (Δ ppm)	$[R_2CH_2\text{-}COO]^-$ *m/z* (Δ ppm)	$[M\text{-}H\text{-}R_1'\text{-}CH{=}C{=}O]^-$ *m/z* (Δ ppm)	$[M\text{-}H\text{-}R_2'\text{-}CH{=}C{=}O]^-$ *m/z* (Δ ppm)	Present in CSL Mix	Cells	Group
PC-P(20:1/20:4)	7,28 (6)	$[M\text{-}CH_3]^-$	804.591 (2)	168.0425 (2)	/	303.2328 (0)	/	518.3575 (8)	No	Yes	2
PC(14:0/16:1)	5,38 (6)	$[M\text{+}OAc]^-$	762.5259 (6)	168.0431 (2)	227.2016 (0)	253.216 (5)	478.2823 (24)	452.2778 (1)	No	Yes	2
PC(14:0/16:1)	5,41 (6)	$[M\text{-}CH_3]^-$	688.4888 (6)	168.0432 (3)	227.2015 (0)	253.2182 (4)	478.2838 (22)	452.2791 (2)	No	Yes	2
PC(16:0/14:0)	5,89 (2)	$[M\text{-}CH_3]^-$	690.507 (2)	168.043 (2)	255.2322 (3)	227.2008 (3)	/	480.3028 (14)	No	Yes	2
PC(16:0/16:0) **	6,45 (0)	$[M\text{-}CH_3]^-$	718.539 (1)	168.0386 (24)	255.2327 (1)	255.2327 (1)	480.316 (12)	480.316 (13)	Yes	Yes	1
PC(16:0/18:1) **	6,55 (0)	$[M\text{-}CH_3]^-$	744.554 (2)	168.041 (10)	255.2321 (3)	281.2484 (1)	506.3283 (4)	480.3126 (6)	Yes	Yes	1
PC(16:0/18:2) **	6,08 (2)	$[M\text{-}CH_3]^-$	742.536 (5)	168.0393 (20)	253.2167 (2)	281.2484 (1)	506.3279 (4)	478.292 (4)	Yes	Yes	1
PC(16:1/18:2)	5,82 (2)	$[M\text{-}CH_3]^-$	740.524 (0)	168.0415 (7)	253.2152 (8)	279.2307 (8)	504.2863 (51)	478.2919 (4)	No	Yes	2
PC(16:1/22:0)	7,89 (8)	$[M\text{-}CH_3]^-$	800.619 (1)	168.0444 (10)	253.2175 (1)	339.3254 (5)	564.3956 (14)	478.2946 (1)	No	Yes	2
PC(17:0/17:0) *	6,96 (2)	$[M\text{-}CH_3]^-$	746.571 (0)	168.0439 (7)	269.248 (2)	269.248 (2)	494.3306 (9)	494.3306 (11)	Yes	No	1
PC(18:0/18:1) **	6,99 (1)	$[M\text{-}CH_3]^-$	772.586 (1)	168.0455 (17)	283.2621 (8)	281.2478 (3)	506.3136 (28)	508.3333 (15)	Yes	Yes	1
PC(18:0/18:2) **	6,6 (1)	$[M\text{-}CH_3]^-$	770.57 (2)	168.0467 (24)	283.2637 (2)	279.2307 (8)	504.3153 (10)	506.325 (15)	Yes	Yes	1
PC(18:0/20:4) **	6,62 (3)	$[M\text{-}CH_3]^-$	794,568 (4)	168.0398 (17)	283.2637 (2)	303.2326 (1)	528.3065 (7)	508.3417 (1)	Yes	Yes	1
PC(18:2/18:1)	6,23 (2)	$[M\text{-}CH_3]^-$	768.554 (2)	168.0425 (1)	279.2316 (5)	281.2478 (3)	506.3361 (18)	504.3062 (7)	No	Yes	2
PC(20:1/20:1) *	7,42 (0)	$[M\text{-}CH_3]^-$	826.629 (6)	168.0425 (1)	309.2802 (0)	309.2802 (0)	534.3513 (10)	534.3513 (10)	Yes	No	1
PC(22:1/22:1) *	8,14 (1)	$[M\text{-}CH_3]^-$	882.69 (8)	168.0452 (15)	337.3107 (2)	337.3107 (2)	562.3857 (6)	562.3857 (4)	Yes	No	1
PC(P-18:0/20:4) *	6,75 (0)	$[M\text{-}CH_3]^-$	778.574 (7)	168.0365 (7)	/	303.2324 (2)	/	492.3476 (4)	Yes	Yes	1
PE 18:0/18:0	7,47 (3)	$[M\text{-}H]^-$	746.564 (5)	140.0148 (3)	283.264 (1)	283.264 (1)	480.3032 (13)	480.3032 (14)	No	Yes	2
PE-O(16:0/20:4)	6,53 (2)	$[M\text{-}H]^-$	724.5238 (7)	140.0113 (0)	/	303.2321 (3)	/	438.2859 (29)	No	Yes	2
PE-O(18:0/20:5)	6,5 (1)	$[M\text{-}H]^-$	750.5413 (2)	140.0113 (14)	/	301.2158 (5)	/	466.313 (33)	No	Yes	2
PE-O(18:0/22:6)	6,9 (3)	$[M\text{-}H]^-$	776.549 (12)	140.0114 (15)	/	327.2315 (4)	/	466.3176 (23)	No	Yes	2

Name	t_R min (Δ%)	Adduct	Precursor ion *m/z* (Δ ppm)	Polar head group *m/z* (Δ ppm)	$[R_1CH_2\text{-}COO]^-$ *m/z* (Δ ppm)	$[R_2CH_2\text{-}COO]^-$ *m/z* (Δ ppm)	$[M\text{-}H\text{-}R_1'\text{-}CH{=}C{=}O]^-$ *m/z* (Δ ppm)	$[M\text{-}H\text{-}R_2'\text{-}CH{=}C{=}O]^-$ *m/z* (Δ ppm)	Present in CSL Mix	Present in Cells	Group
PE-P(16:0/16:0)	6,85 (2)	[M-H]-	674.511 (3)	140.0181 (49)	/	255.2326 (1)	/	436.2837 (2)	No	Yes	2
PE-P(16:0/16:1)	6,4 (0)	[M-H]-	672.494 (5)	140.0056 (40)	/	253.2167 (2)	/	436.2825 (1)	No	Yes	2
PE-P(16:0/16:1)	6,43 (0)	[M-H]-	674.503 (5)	140.0103 (7)	/	253.2168 (2)	/	438.3012 (6)	No	Yes	2
PE-P(16:0/20:4)	6,45 (0)	[M-H]-	724.518 (5)	140.0113 (0)	/	303.2321 (3)	/	438.3012 (6)	No	Yes	2
PE-P(16:0/22:6)	6,29 (1)	[M-H]-	746.51 (4)	140.0121 (6)	/	327.2325 (1)	/	436.2824 (1)	No	Yes	2
PE-P(17:1/18:1)	6,71 (2)	[M-H]-	712.528 (1)	140.0101 (8)	/	281.2487 (0)	/	448.287 (9)	No	Yes	2
PE-P(18:0/16:0)	7,3 (1)	[M-H]-	702.543 (2)	140.0069 (31)	/	255.2325 (2)	/	464.3063 (17)	No	Yes	2
PE-P(18:0/20:3)	7,07 (2)	[M-H]-	752.558 (3)	140.0103 (7)	/	305.248 (2)	/	464.3109 (7)	No	Yes	2
PE-P(18:0/20:4) **	6,87 (0)	[M-H]-	750.544 (1)	140.0108 (3)	/	303.2329 (0)	/	464.314 (1)	Yes	Yes	1
PE-P(18:0/22:3)	7,55 (2)	[M-H]-	780.586 (7)	140.0103 (7)	/	333.2785 (5)	/	464.3281 (30)	No	Yes	2
PE-P(18:0/22:6) **	6,75 (0)	[M-H]-	774.544 (1)	140.0119 (5)	/	327.2328 (0)	/	464.3154 (2)	Yes	Yes	1
PE-P(18:0/24:5)	7,32 (2)	[M-H]-	804.5884 (4)	140.0169 (40)	/	357.2797 (1)	/	464.3133 (2)	No	Yes	2
PE-P(18:1/16:1)	6,49 (1)	[M-H]-	698.512 (2)	140.0169 (40)	/	253.2164 (3)	/	462.2965 (4)	No	Yes	2
PE-P(18:1/18:1)	6,93 (2)	[M-H]-	726.543 (2)	140.0097 (11)	/	281.2496 (3)	/	462.2979 (1)	No	Yes	2
PE-P(18:1/22:3)	7,18 (2)	[M-H]-	778.574 (2)	140.0087 (18)	/	333.2797 (1)	/	462.2995 (2)	No	Yes	2
PE-P(18:1/22:5)	6,53 (3)	[M-H]-	774.5427 (2)	140.0119 (5)	/	329.2481 (2)	/	462.2966 (4)	No	Yes	2
PE-P(18:1/22:6)	6,36 (1)	[M-H]-	772.527 (10)	140.0094 (54)	/	327.2336 (2)	/	462.3001 (9)	No	Yes	2
PE(14:0/14:0) **	5,45 (1)	[M-H]-	634.445 (1)	140.0111 (3)	227.2012 (2)	227.2012 (2)	424.2642 (41)	424.2642 (40)	Yes	Yes	1
PE(16:0/16:0) **	6,57 (3)	[M-H]-	690.503 (8)	140.0122 (3)	255.2319 (4)	255.2319 (4)	452.2769 (3)	452.2769 (5)	Yes	Yes	1
PE(16:0/16:1)	6,15 (1)	[M-H]-	688.491 (2)	140.0101 (8)	253.217 (1)	255.234 (4)	452.2795 (3)	450.255 (15)	No	Yes	2
PE(16:0/16:2)	5,96 (2)	[M-H]-	686.473 (6)	140.0129 (12)	251.2009 (3)	255.2316 (5)	452.2851 (15)	448.2419 (14)	No	Yes	2

Name	t_R min (Δ%)	Adduct	Precursor ion *m/z* (Δ ppm)	Polar head group *m/z* (Δ ppm)	$[R_1CH_2\text{-}COO]^-$ *m/z* (Δ ppm)	$[R_2CH_2\text{-}COO]^-$ *m/z* (Δ ppm)	$[M\text{-}H\text{-}R_1'\text{-}CH{=}C{=}O]^-$ *m/z* (Δ ppm)	$[M\text{-}H\text{-}R_2'\text{-}CH{=}C{=}O]^-$ *m/z* (Δ ppm)	Present in CSL Mix	Cells	Group
PE(16:0/20:4)	6,2 (1)	$[M-H]^-$	738.5054 (4)	140.0117 (3)	303.2328 (0)	255.2331 (0)	452.2776 (1)	500.2738 (9)	No	Yes	2
PE(17:0/20:4) **	6,43 (1)	$[M-H]^-$	752.521 (4)	140.0106 (3)	303.2316 (4)	269.2488 (0)	466.2955 (4)	500.2707 (15)	Yes	Yes	1
PE(17:1/16:1)	5,95 (2)	$[M-H]^-$	700.491 (2)	140.0123 (3)	253.2163 (4)	267.2324 (2)	464.2816 (7)	450.2749 (26)	No	Yes	2
PE(18:0/18:1) **	7,07 (2)	$[M-H]^-$	744.554 (2)	140.0114 (1)	281.2476 (4)	283.2631 (4)	480.3136 (8)	478.2919 (7)	Yes	Yes	1
PE(18:0/20:3)	6,88 (0)	$[M-H]^-$	768.555 (1)	140.0119 (5)	305.2488 (0)	283.2637 (2)	480.3105 (2)	502.289 (11)	No	Yes	2
PE(18:0/20:4) **	6,67 (1)	$[M-H]^-$	766.541 (2)	140.0118 (3)	303.2336 (2)	283.2643 (0)	480.3101 (1)	500.2749 (7)	Yes	Yes	1
PE(18:0/22:6)	6,51 (0)	$[M-H]^-$	790.537 (3)	140.0116 (3)	327.2318 (3)	283.2555 (31)	480.309 (1)	524.2709 (31)	No	Yes	2
PE(18:1/16:0)	6,61 (1)	$[M-H]^-$	716.522 (3)	140.0116 (3)	281.2485 (1)	255.2327 (1)	452.278 (0)	478.2945 (1)	No	Yes	2
PE(18:1/16:1) **	6,18 (1)	$[M-H]^-$	714.507 (2)	140.0107 (4)	253.2156 (7)	281.247 (6)	478.2882 (12)	450.2696 (12)	Yes	Yes	1
PE(18:1/20:4)	6,27 (1)	$[M-H]^-$	764.522 (3)	140.0161 (35)	303.2323 (2)	281.2474 (4)	478.2914 (5)	500.2642 (31)	No	Yes	2
PE(22:1/18:1)	7,57 (0)	$[M-H]^-$	798.6018 (1)	140.0115 (3)	281.2481 (2)	337.3108 (2)	534.3564 (0)	478.2946 (0)	No	Yes	2
PE(24:0/18:1)	8,24 (0)	$[M-H]^-$	828.649 (1)	140.0158 (33)	281.2475 (4)	367.3559 (7)	564.4045 (1)	478.2855 (23)	No	Yes	2
PE(26:0/18:1)	8,53 (2)	$[M-H]^-$	856.682 (1)	140.011 (2)	281.2477 (3)	395.3876 (6)	592.4298 (9)	478.2914 (10)	No	Yes	2
PE(26:1/18:1)	8,2 (2)	$[M-H]^-$	854.6614 (5)	140.0068 (32)	281.2474 (4)	393.3729 (3)	590.4191 (1)	478.2911 (9)	No	Yes	2
PG(14:0/14:0) *	4,43 (4)	$[M-H]^-$	665.437 (6)	227.0399 (15)	227.2007 (4)	227.2007 (4)	455.2441 (5)	455.2441 (5)	Yes	No	1
PG(16:0/16:0) **	5,59 (3)	$[M-H]^-$	721,498 (7)	227,0305 (9)	255,233 (0)	255,233 (0)	483,2704 (6)	483,2704 (6)	Yes	Yes	1
PG(16:0/18:1) **	5,59 (0)	$[M-H]^-$	747,518 (1)	227,0343 (8)	255,2332 (1)	281,2479 (3)	509,3002 (22)	483,2786 (11)	Yes	Yes	1
PG(17:0/17:0) *	6,02 (0)	$[M-H]^-$	749,5303 (6)	227,0253 (32)	269,2478 (3)	269,2478 (3)	497,285 (8)	497,285 (8)	Yes	No	1
PG(18:0/18:0) **	6,53 (1)	$[M-H]^-$	777,5649 (2)	227,0348 (10)	283,263 (5)	283,263 (5)	511,3052 (1)	511,3052 (1)	Yes	Yes	1
PG(18:0/18:2) **	5,73 (0)	$[M-H]^-$	773,5302 (6)	227,029 (15)	283,2634 (3)	279,2321 (3)	507,2715 (3)	511,3032 (3)	Yes	Yes	1
PG(18:1/18:0) **	6,14 (0)	$[M-H]^-$	775,547 (4)	227,0293 (14)	281,2475 (4)	283,2624 (7)	511,3036 (2)	509,2921 (6)	Yes	Yes	1

**Name**	**t_R min (Δ%)**	**Adduct**	**Precursor ion *m/z* (Δ ppm)**	**Polar head group *m/z* (Δ ppm)**	**$[R_1CH_2\text{-}COO]^-$ *m/z* (Δ ppm)**	**$[R_2CH_2\text{-}COO]^-$ *m/z* (Δ ppm)**	**$[M\text{-}H\text{-}R_1'\text{-}CH{=}C{=}O]^-$ *m/z* (Δ ppm)**	**$[M\text{-}H\text{-}R_2'\text{-}CH{=}C{=}O]^-$ *m/z* (Δ ppm)**	**Present in CSL Mix**	**Cells**	**Group**
PG(18:2/18:2)	4,84 (0)	$[M-H]^-$	769,5 (4)	227,0399 (33)	279,2328 (1)	279,2328 (1)	507,2683 (10)	507,2683 (10)	Yes	No	1
PI(16:0/16:0) *	5,52 (1)	$[M-H]^-$	809.515 (6)	241.0123 (2)	255.2309 (8)	255.2307 (9)	/	553.279 (0)	Yes	Yes	1
PI(16:0/18:0)	5,75 (4)	$[M-H]^-$	837.55 (1)	241.0099 (8)	283.2642 (1)	255.2315 (6)	/	581.2972 (22)	No	Yes	2
PI(16:0/20:2)	5,69 (2)	$[M-H]^-$	861.54 (13)	241.0107 (4)	255.2329 (0)	307.2659 (5)	/	553.2758 (5)	No	Yes	2
PI(16:0/20:3)	5,43 (1)	$[M-H]^-$	859.5326 (3)	241.0105 (5)	255.2364 (13)	305.2485 (0)	/	553.2773 (3)	No	Yes	2
PI(18:0/18:0) *	6,46 (0)	$[M-H]^-$	865.5777 (5)	241.0123 (2)	283.2631 (4)	283.2631 (4)	/	581.3075 (4)	Yes	Yes	1
PI(18:0/20:4) *	5,68 (1)	$[M-H]^-$	885.5487 (3)	241.0077 (17)	283.2625 (7)	303.2278 (17)	/	()	Yes	Yes	1
PI(18:0/20:5)	5,23 (2)	$[M-H]^-$	883.534 (1)	241.0099 (8)	283.2633 (4)	301.2163 (3)	/	581.3004 (17)	No	Yes	2
PI(18:0/22:4)	6,01 (3)	$[M-H]^-$	913.58 (3)	241.0114 (2)	283.261 (12)	331.2582 (19)	/	581.3016 (15)	No	Yes	2
PI(18:0/22:6)	5,56 (1)	$[M-H]^-$	909.5472 (4)	241.0126 (3)	283.264 (1)	327.2403 (23)	/	581.3021 (14)	No	Yes	2
PI(18:1/16:0)	5,58 (1)	$[M-H]^-$	835.532 (4)	241.0113 (2)	255.2318 (5)	281.2462 (9)	/	553.2708 (14)	No	Yes	2
PI(18:1/18:0)	6,1 (1)	$[M-H]^-$	863.562 (5)	241.0106 (5)	281.2485 (1)	283.2628 (6)	/	579.2969 (4)	No	Yes	2
PI(18:1/20:5)	4,8 (4)	$[M-H]^-$	881.517 (3)	241.0128 (4)	281.2485 (1)	301.2221 (16)	/	579.2926 (3)	No	Yes	2
PS(16:0/16:0) **	5,6 (4)	$[M-H]^-$	734.494 (1)	152.9961 (2)	255.2332 (1)	255.2332 (1)	409.2357 (6)	391.2234 (4)	Yes	Yes	1
PS(16:0/16:1)	5,14 (2)	$[M-H]^-$	732.4774 (2)	152.9962 (3)	255.2334 (2)	253.2128 (18)	409.2333 (0)	391.2228 (2)	No	Yes	2
PS(16:0/18:0)	6 (0)	$[M-H]^-$	762.5073 (25)	152.9952 (3)	255.2332 (1)	283.2647 (1)	409.2364 (8)	391.2247 (7)	No	Yes	2
PS(16:0/18:1)	5,68 (1)	$[M-H]^-$	760.511 (1)	152.9944 (9)	255.2322 (3)	281.2505 (7)	409.2304 (7)	391.224 (5)	No	Yes	2
PS(16:0/18:2) **	5,31 (1)	$[M-H]^-$	758.493 (2)	152.9957 (0)	255.232 (4)	279.2318 (4)	409.2303 (7)	391.2258 (10)	Yes	Yes	1
PS(16:0/20:3)	5,4 (1)	$[M-H]^-$	784.508 (3)	152.9964 (4)	255.2317 (5)	305.2419 (22)	409.2351 (4)	391.2242 (6)	No	Yes	2
PS(16:0/20:5)	4,8 (2)	$[M-H]^-$	780.4784 (1)	152.9968 (7)	255.2355 (10)	301.2146 (9)	409.239 (14)	391.2214 (2)	No	Yes	2
PS(17:0/18:1)	5,91 (0)	$[M-H]^-$	774.5253 (1)	152.9987 (19)	269.2428 (22)	281.2535 (17)	423.256 (17)	405.244 (16)	No	Yes	2

Name	t_R min (Δ%)	Adduct	Precursor ion *m/z* (Δ ppm)	Polar head group *m/z* (Δ ppm)	$[R_1CH_2\text{-}COO]^-$ *m/z* (Δ ppm)	$[R_2CH_2\text{-}COO]^-$ *m/z* (Δ ppm)	$[M\text{-}H\text{-}R_1'\text{-}CH{=}C{=}O]^-$ *m/z* (Δ ppm)	$[M\text{-}H\text{-}R_2'\text{-}CH{=}C{=}O]^-$ *m/z* (Δ ppm)	Present in CSL Mix	Cells	Group
PS(18:0/18:0)	6,6 (1)	$[M\text{-}H]^-$	790.555 (3)	152.9961 (2)	283.2641 (1)	283.2641 (1)	/	419.2538 (1)	No	Yes	2
PS(18:0/18:1) **	6,22 (1)	$[M\text{-}H]^-$	788.539 (3)	152.9955 (1)	283.2632 (4)	281.2465 (8)	437.2654 (2)	419.2554 (5)	Yes	Yes	1
PS(18:0/20:2)	6,3 (1)	$[M\text{-}H]^-$	814.552 (7)	152.9957 (0)	283.2645 (0)	307.2609 (11)	437.2667 (5)	419.2568 (8)	No	Yes	2
PS(18:0/20:3)	6,06 (1)	$[M\text{-}H]^-$	812.542 (0)	152.9988 (20)	283.2633 (4)	305.2504 (6)	437.2658 (3)	419.2561 (6)	No	Yes	2
PS(18:0/20:4)	5,73 (2)	$[M\text{-}H]^-$	810.527 (1)	152.9984 (17)	283.2622 (8)	303.2292 (12)	437.2764 (27)	419.2564 (7)	No	Yes	2
PS(18:1/20:0)	6,77 (1)	$[M\text{-}H]^-$	816.576 (4)	152.997 (8)	281.2491 (2)	311.2913 (14)	435.2396 (22)	417.237 (2)	No	Yes	2
PS(18:1/20:1)	6,2 (3)	$[M\text{-}H]^-$	814.5555 (2)	152.9948 (6)	281.246 (9)	309.2781 (6)	435.2266 (51)	417.2387 (2)	No	Yes	2
PS(18:1/22:0)	7,17 (2)	$[M\text{-}H]^-$	844.6059 (2)	152.9968 (7)	281.2457 (11)	339.3269 (1)	435.248 (2)	417.2322 (13)	No	Yes	2
PS(18:1/22:1)	6,73 (3)	$[M\text{-}H]^-$	842.592 (4)	152.9959 (1)	281.2465 (8)	337.3094 (6)	435.2499 (2)	417.2379 (0)	No	Yes	2
PS(18:1/24:1)	7,14 (5)	$[M\text{-}H]^-$	870.6226 (3)	152.9946 (7)	281.2482 (2)	365.3359 (19)	435.2294 (45)	417.2436 (14)	No	Yes	2

Table S3 B. Annotation of lipid species by MS/MS experiment of ceramide and hexosylceramide fragmentation. Fragmentations were acquired from the precursor ion. Annotation of product ions according to Anh *et al.*, *Anal Chem*, 1993. Thanks to accurate *m/z* measurement, MS/MS data inspection and retention time analysis, lipids annotated in HCE cells were assigned to group 1 or 2 according to the guidelines provided by the minimum reporting standards of the Metabolomics Standards Initiative. Annotated lipids labelled with * are indicative of standard lipids whereas those labelled with ** are indicative of standard lipids also found in HCE cells. CSL Mix = Commercial Standard Lipid Mixture

Name	t_R min (Δ%)	Adduct	Precursor ion *m/z* (Δ ppm)	S *m/z* (Δ ppm)	T *m/z* (Δ ppm)	P *m/z* (Δ ppm)	$[M-2H_2O-H]^-$ *m/z* (Δ ppm)	Neutral loss Th (Δ ppm)	Present in CSL Mix	Present in Cells	Group
Cer(d18:1/12:0) *	5,36 (0)	$[M-H]^-$	480.441 (3)	240.1962 (4)	224.2018 (4)	237.222 (1)	432.4198 (5)	/	Yes	No	1
Cer(d18:1/14:0) **	5,97 (1)	$[M-H]^-$	508.472 (4)	268.2277 (4)	252.2327 (5)	237.2215 (3)	460.4509 (6)	/	Yes	Yes	1
Cer(d18:1/16:0) **	6,55 (0)	$[M-H]^-$	536.503 (5)	296.2584 (6)	280.2646 (3)	237.2218 (2)	488.4837 (2)	/	Yes	Yes	1
Cer(d18:1/18:0) **	7,08 (0)	$[M-H]^-$	564.5368 (0)	324.2936 (7)	308.2932 (12)	237.2244 (9)	516.5209 (9)	/	Yes	Yes	1
Cer(d18:1/22:0)	7,95 (1)	$[M-H]^-$	620.596 (6)	380.3503 (10)	364.3562 (10)	237.2222 (0)	572.5817 (5)	/	No	Yes	2
Cer(d18:1/23:0)	8,15 (1)	$[M-H]^-$	634.612 (5)	394.3662 (9)	378.3747 (2)	237.2279 (24)	586.5993 (8)	/	No	Yes	2
Cer(d18:1/23:1)	7,76 (1)	$[M-H]^-$	632.596 (6)	392.3658 (30)	376.3588 (2)	237.2206 (7)	584.5713 (13)	/	No	Yes	2
Cer(d18:1/24:0)	8,29 (1)	$[M-H]^-$	648.627 (6)	408.3816 (10)	392.3868 (11)	237.2209 (6)	600.6014 (15)	/	No	Yes	2
Cer(d18:1/24:1) **	7,86 (0)	$[M-H]^-$	646.611 (7)	406.3696 (1)	390.3735 (5)	237.2217 (2)	598.5919 (5)	/	Yes	Yes	1
Cer(d18:1/26:0)	8,6 (2)	$[M-H]^-$	676.6578 (7)	436.4076 (21)	420.4243 (4)	237.221 (5)	628.6432 (2)	/	No	Yes	2
Cer(d18:1/26:1)	8,26 (1)	$[M-H]^-$	674.645 (2)	434.3957 (13)	418.4064 (1)	237.2191 (13)	626.6234 (4)	/	No	Yes	2
Cer(d18:2/16:0)	6,14 (2)	$[M-H]^-$	534.488 (3)	296.2581 (7)	280.2633 (8)	235.2048 (8)	486.4634 (12)	/	No	Yes	2
Cer(d18:2/18:0)	6,71 (2)	$[M-H]^-$	562.518 (6)	324.2908 (2)	308.2961 (2)	235.2118 (22)	514.4961 (9)	/	No	Yes	2
Cer(d18:2/22:0)	7,58 (1)	$[M-H]^-$	618.579 (8)	380.3622 (21)	364.3581 (4)	235.2039 (11)	570.5594 (7)	/	No	Yes	2

Name	t_R min (Δ%)	Adduct	Precursor ion *m/z* (Δ ppm)	S *m/z* (Δ ppm)	T *m/z* (Δ ppm)	P *m/z* (Δ ppm)	$[M\text{-}2H_2O\text{-}H]^-$ *m/z* (Δ ppm)	Neutral loss Th (Δ ppm)	Present in CSL Mix	Cells	Group
Cer(d18:2/24:1)	7,61 (1)	$[M\text{-}H]^-$	644.595 (7)	406.3698 (0)	390.3722 (8)	235.2063 (1)	596.577 (3)	/	No	Yes	2
Cer(d18:2/26:1)	7,96 (1)	$[M\text{-}H]^-$	672.626 (7)	434.3995 (4)	418.4026 (10)	235.2059 (3)	624.6091 (2)	/	No	Yes	2
GalCer(d18:1/18:0) **	6,62 (0)	$[M\text{-}H]^-$	726.5867 (4)	324.2835 (24)	308.2943 (8)	237.2231 (3)	516.5104 (11)	194.0733 (26)	Yes	Yes	1
GalCer(d18:1/24:1) **	7,48 (1)	$[M\text{-}H]^-$	808.666 (2)	406.359 (27)	390.3723 (8)	237.2216 (3)	598.6009 (10)	194.0805 (23)	Yes	Yes	1
HexCer(18:0/24:1)	7,54 (0)	$[M\text{-}H]^-$	810.675 (11)	406.3801 (25)	390.3725 (7)	239.2325 (23)	600.5909 (32)	194.074 (24)	No	Yes	2
HexCer(18:1/16:0)	6,14 (0)	$[M\text{-}H]^-$	698.5552 (4)	296.2517 (28)	280.2648 (2)	237.219 (14)	488.4813 (7)	194.086 (40)	No	Yes	2
HexCer(18:1/22:0)	7,58 (0)	$[M\text{-}H]^-$	782.649 (4)	380.3525 (4)	364.3595 (0)	237.2228 (2)	572.5879 (16)	194.062 (84)	No	Yes	2
HexCer(18:1/23:0)	7,77 (0)	$[M\text{-}H]^-$	796.6635 (6)	394.3712 (3)	378.369 (17)	/	586.6019 (12)	194.0825 (70)	No	Yes	2
HexCer(18:1/24:0)	7,93 (0)	$[M\text{-}H]^-$	810.679 (6)	408.3934 (19)	392.3832 (20)	237.2208 (6)	600.5989 (19)	194.0877 (74)	No	Yes	2
HexCer(18:1/26:1)	7,91 (1)	$[M\text{-}H]^-$	836.6939 (6)	434.3925 (20)	418.4164 (23)	/	626.6271 (2)	194.0925 (73)	No	Yes	2
LacCer(18:1/24:0)		[M-H]-	972.7319 (5)	408.3755 (25)	392.3898 (3)	/	/	/	No	Yes	2
LacCer(18:1/24:1)		$[M\text{-}H]^-$	970.72 (1)	406.3865 (41)	390.3661 (24)	/	/	/	No	Yes	2

Table S3 C. Annotation of lipid species by MS/MS experiment of sphingomyelin fragmentation. Fragmentations were acquired from the precursor ion. Annotation of product ions according to Anh *et al.*, *Anal Chem*, 1993. Thanks to accurate *m/z* measurement, MS/MS data inspection and retention time analysis, lipids annotated in HCE cells were assigned to group 1 or 2 according to the guidelines provided by the minimum reporting standards of the Metabolomics Standards Initiative. Annotated lipids labelled with * are indicative of standard lipids whereas those labelled with ** are indicative of standard lipids also found in HCE cells. Mix = Commercial Standard Lipid Mixture

**Name**	**t_R**	**Adduct**	**Precursor ion**	**Polar head group**	**O**	**Present in**		
	**min (Δ%)**		***m/z* (Δ ppm)**	***m/z* (Δ ppm)**	***m/z* (Δ ppm)**	**CSL Mix**	**Cells**	**Group**
SM(d18:0/16:0)	6.12 (5)	$[M-CH_3]^-$	689.5575 (5)	168.0432 (3)	451.32 (22)	No	Yes	2
SM(d18:1:22:0)	7.43 (0)	$[M-CH_3]^-$	771.636 (5)	168.0426 (1)	449.3294 (39)	No	Yes	2
SM(d18:1/12:0) *	4.57 (0)	$[M-CH_3]^-$	631.4805 (3)	168.0421 (4)	449.3285 (37)	Yes	No	1
SM(d18:1/14:0) **	5.23 (1)	$[M-CH_3]^-$	659.5096 (7)	168.0415 (7)	449.323 (25)	Yes	Yes	1
SM(d18:1/16:0) **	5.89 (0)	$[M-CH_3]^-$	687.5421 (5)	168.0414 (8)	449.3243 (28)	Yes	Yes	1
SM(d18:1/16:1)	5.38 (2)	$[M-CH_3]^-$	685.5265 (5)	168.0436 (5)	449.3138 (5)	No	Yes	2
SM(d18:1/18:0) **	6.45 (0)	$[M-CH_3]^-$	715.5738 (4)	168.044 (8)	449.323 (25)	Yes	Yes	1
SM(d18:1/18:1)	6.14 (4)	$[M-CH_3]^-$	689.5595 (3)	168.044 (8)		No	Yes	2
SM(d18:1/22:0)	7.46 (0)	$[M-CH_3]^-$	771.636 (5)	168.0426 (1)	449.3294 (39)	No	Yes	2
SM(d18:1/24:0) **	7.83 (0)	$[M-CH_3]^-$	799.6652 (7)	168.0427 (0)	449.3354 (53)	Yes	Yes	1
SM(d18:1/24:1) **	7.42 (0)	$[M-CH_3]^-$	797.6523 (4)	168.0438 (6)	449.3162 (10)	Yes	Yes	1

Figure S1. (A) Extracted ion chromatograms of *m/z* values corresponding to precursor ions of standard phospholipids containing two palmitoyl moieties at sn_1 and sn_2 positions. (B) Analytical features of each precursor ion.

A

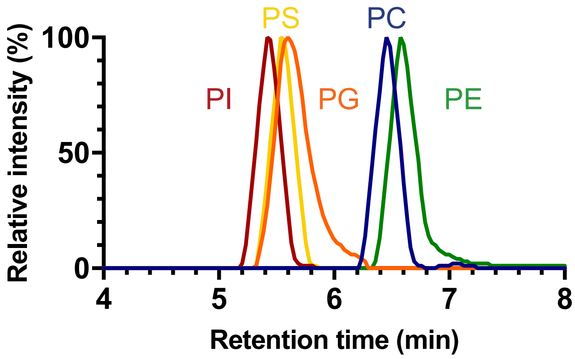

B

Lipid species	t_R	*m/z*	Adduct
PI (16:0/16:0)	5.52	809.5155	$[M-H]^-$
PS (16:0/16:0)	5.60	734.4959	$[M-H]^-$
PG (16:0/16:0)	5.65	721.4998	$[M-H]^-$
PC (16:0/16:0)	6.45	718.5373	$[M-CH_3]^-$
PE (16:0/16:0)	6.57	690.5060	$[M-H]^-$

Figure S2. (A) MN of PS subclass (B) MS/MS spectra of five different PS [M-H]$^-$ ions. (C) Fragmentation scheme of PS ions. Table: exact mass measurement of diagnostic fragment ions of the same deprotonated PS molecules.

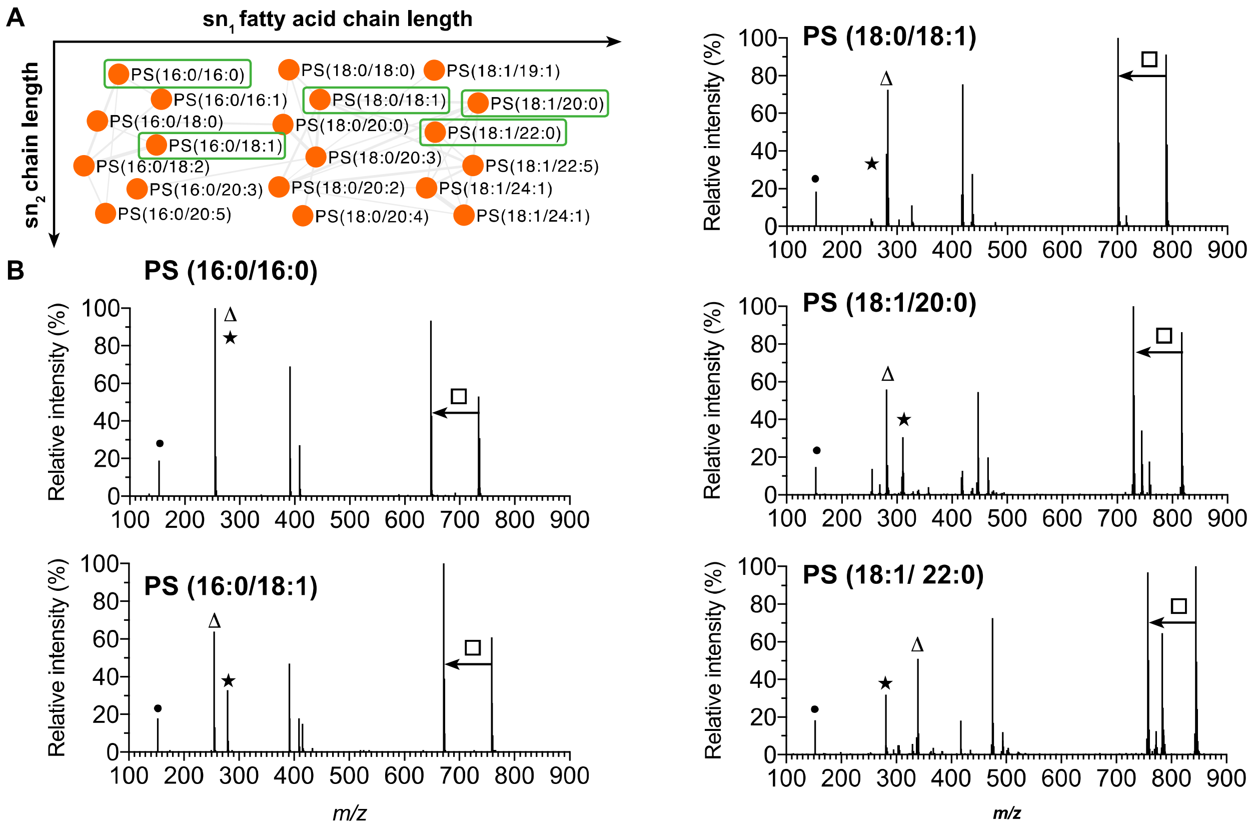

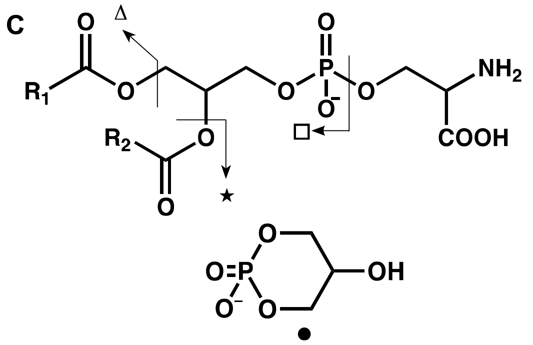

Species	● *m/z* (ppm)	Δ *m/z* (ppm)	★ *m/z* (ppm)	□ Th (ppm)
PS (16:0/16:0)	152.9944 (9)	255.2322 (3)	281.2505 (7)	87.033 (5)
PS(16:0/18:1)	152.9961 (2)	255.2332 (1)	255.2332 (1)	87.0328 (3)
PS (18:0/18:1)	152.9955 (1)	283.2632 (4)	281.2465 (8)	87.0322 (4)
PS (18:1/20:0)	152.997 (8)	281.2491 (2)	311.2943 (5)	87.0325 (1)

Figure S3. (A) MN in PI subclass (B) Fragmentation scheme of PI ions (C) MS/MS spectra of four representative PI deprotonated molecules. Table: exact mass measurement of the diagnostic fragment ions of the same deprotonated PS molecules.

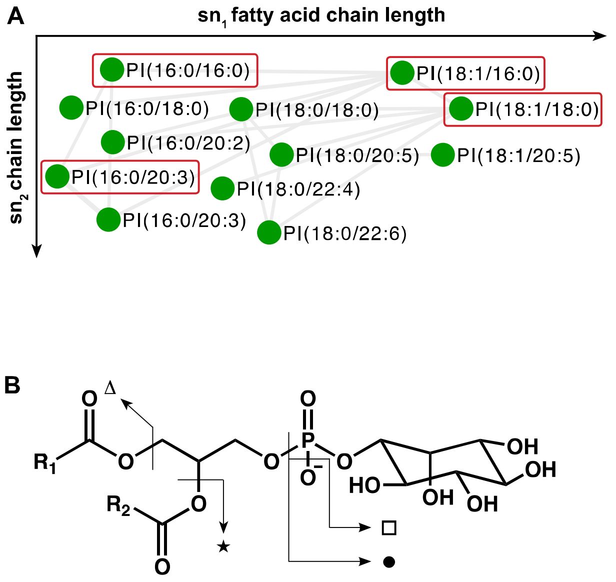

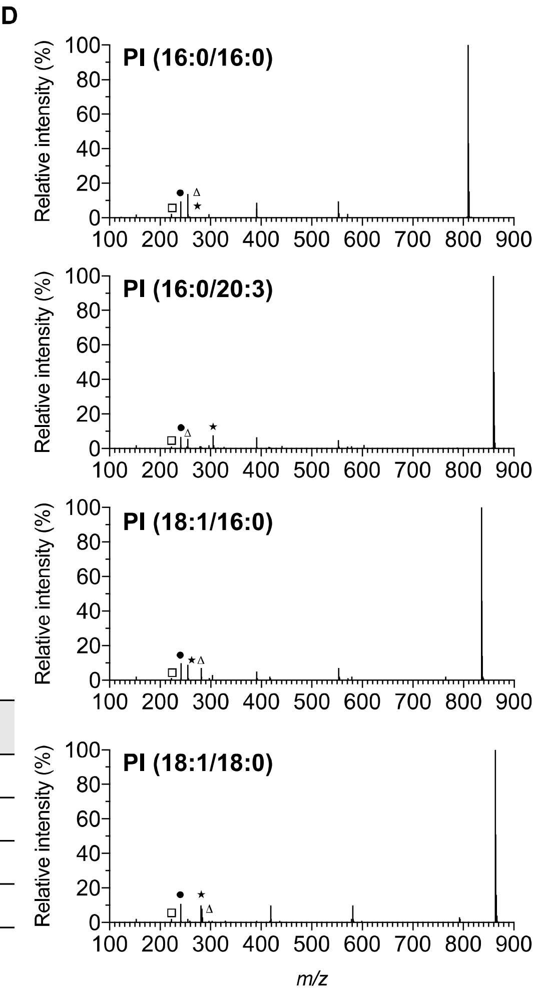

Species	□ *m/z* (ppm)	● *m/z* (ppm)	Δ *m/z* (ppm)	★ *m/z* (ppm)
PI (16:0/16:0)	222.9991 (9)	241.0123 (2)	255.2309 (8)	255.2327 (1)
PI (16:0/20:3)	223.003 (8)	241.0116 (1)	255.2352 (9)	305.2485 (0)
PI (18:1/16:0)	223.003 (8)	241.0113 (2)	281.2462 (9)	255.2318 (5)
PI (18:1/18:0)	222.9999 (6)	241.0118 (0)	281.2468 (7)	283.2634 (3)

Figure S4. Annotation of MS/MS spectra of commercial standard lipids using LipidBlast library (A) MN corresponding to commercial standard lipids. Green nodes correspond to lipids successfully annotated using LipidBlast library while red nodes correspond to lipids for which annotation were not possible with LipidBlast. (B) Percentage of lipids successfully annotated using LipidBlast according to the commercial standard lipid subclasses. Note that except for SM and HexCer, the percentage of lipids successfully annotated by LipidBlast is higher than 65%. Table: Percentage of parent ions, according to the adducts or in source fragments, successfully annotated using LipidBlast for the commercial standard lipid subclasses.

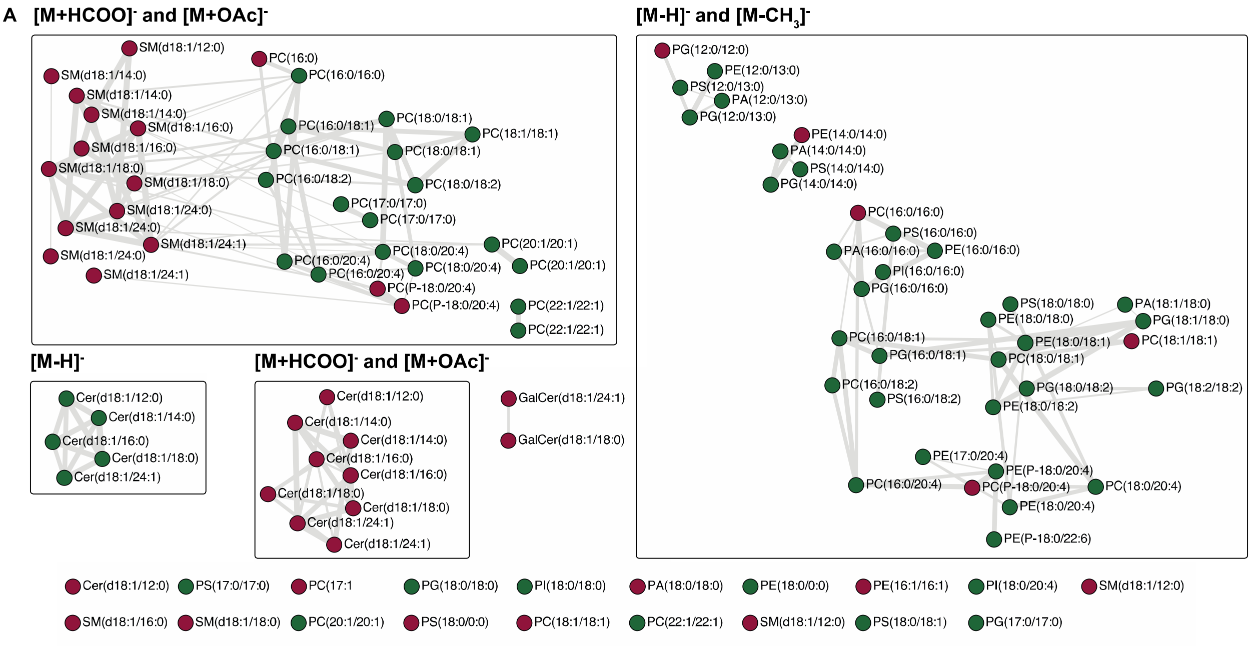

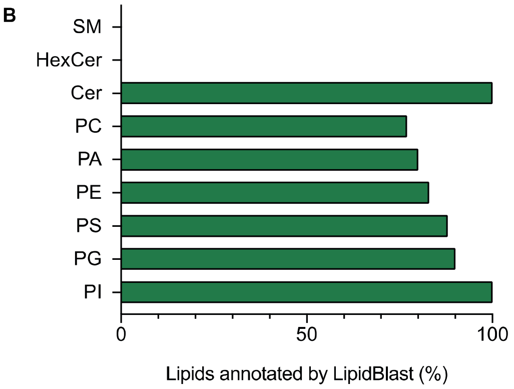

Subclass	[M-H]$^-$	[M+OAc]$^-$	[M+HCOO]$^-$	[M-CH$_3$]$^-$
SM	-	0	0	0
HexCer	0	-	-	-
Cer	100	0	0	-
PC	/	69	69	54
PA	80	-	-	-
PE	83	-	-	-
PS	88	-	-	-
PG	90	-	-	-
PI	100	-	-	-
